# Reduction in Circulating Microplastics in Humans Following Gastrointestinal Sequestration by Chitosan: A Pilot Controlled Study

**DOI:** 10.3390/jox16030092

**Published:** 2026-05-22

**Authors:** Umberto Cornelli, Giovanni Belcaro, Claudio Casella

**Affiliations:** 1 Department of Molecular Pharmacology and Therapeutics, School of Medicine, Loyola University, 2160 1st Ave., Maywood, IL 60660, USA; 2 IRVINE3 Labs and OOLEX Project, University of Pescara-Chieti, 94 Strada Statale 16 Bis 65110 Spoltore, 65110 Pescara, Italy; gianni@belcarocardres.it; 3 Department of Chemistry, University of Pavia, Viale Taramelli 12, 27100 Pavia, Italy; claudio.casella01@universitadipavia.it

**Keywords:** microplastics, chitosan, *Procambarus clarkii*, blood, dietary intervention

## Abstract

Microplastics (MPs) are emerging contaminants that have been detected in human blood and tissues, raising concerns regarding systemic exposure and potential health effects. Internal MP burden mitigation techniques, nevertheless, are yet largely unexplored. We evaluated whether oral administration of chitosan derived from *Procambarus clarkii* (PCC) could reduce circulating MPs in humans via gastrointestinal sequestration in this pilot-controlled study. 11 healthy adults received PCC supplementation (0.8 g/day) for 15 days, while 10 matched controls received a placebo. Using stereomicroscopy, scanning electron microscopy (SEM), and micro-Fourier transform infrared spectroscopy (µFTIR), blood MP concentrations were quantified and characterised according to size, shape, and polymer type. At baseline, MPs were found in every subject. Following PCC supplementation, mean MP concentrations decreased from 1.84 ± 0.28 µg/mL to 1.34 ± 0.20 µg/mL (−26.3%, *p* < 0.01, paired analysis). The control group observed no significant differences. While polymer-resolved analysis consistently indicated reductions across major polymer classes, size-resolved analysis indicated preferential reductions in intermediate particle fractions (11–50 µm). The circulating MPs’ estimated mean residence time (MRT) was 58 ± 28 days. These findings provide preliminary evidence that chitosan-based gastrointestinal sequestration could potentially reduce the systemic MP burden in humans.

## 1. Introduction

Global plastic production exceeded 430 million tons in 2024 [1], resulting in widespread environmental dissemination of microplastics (MPs). MPs have become pervasive contaminants across food, water, and air, originating from the fragmentation of larger plastics and the continuous release from consumer products. Biomonitoring studies now demonstrate that MPs are not limited to external exposure routes but are detectable directly in human biological matrices, including blood [2,3,4] and in virtually all tissues [5] and cerebrospinal fluid, raising concerns about possible health effects [6]. These observations raise essential questions regarding the mechanisms that enable particle uptake, systemic distribution, and tissue persistence. Inhalation and ingestion both contribute to internal exposure, with pulmonary deposition favoring fine particles [7,8] and gastrointestinal uptake mediated by M-cell transcytosis and endocytosis [9].

MPs have been reported to be transported via the bloodstream, which constitutes approximately 6–7% of the total body mass and serves as the primary carrier of oxygen and nutrients [3]. Recent studies indicate MPs’ presence in blood, brain tissue, and cerebrospinal fluid, raising concerns about potential health effects [6], despite early research finding no actual evidence of MPs in human blood [2]. The gastrointestinal system is considered to be the primary pathway of entry into the bloodstream, notwithstanding the observation of pulmonary translocation of inhaled MPs [10,11]. Through atmospheric deposition or medical device abrasion, surgical techniques may also enable direct vascular penetration [11]. Once in circulation, MPs may be cleared through renal filtration or biliary excretion, or they can accumulate in organs, including the liver and spleen, through sinusoids and fenestrated capillaries [2]. Their biological behavior is influenced by size, shape, surface chemistry, and the formation of a biomolecular corona [12]. MPs have been associated with oxidative stress, inflammation, and immune dysregulation, although a causal relationship with disease remains to be established. Elevated blood MP levels have been associated with diabetes, cancer, and cardiovascular disease [6,13,14], and their identification in human atheromas corresponds to higher cardiovascular risk [2]. Only Nanoplastics (NPs, <1 µm) seem to be able to cross the blood–brain barrier (BBB), despite the fact that it restricts exposure to the central nervous system (CNS); large MPs are considered to be physiologically unable of successfully crossing biological barriers and accessing microcirculatory compartments; small MPs (1–10 µm); intestinal absorption is possible but limited; and NPs may be capable of systemic biodistribution and intestinal translocation. [6,15]. The irregular distribution of MPs in blood and the frequent reports of particles larger than 300–500 µm are probably the result of analytical errors. Due to their persistent circulation, MPs bigger than 300–500 µm are incompatible with physiological vascular dimensions and capillary transit. These sizes are ex vivo aggregates or environmental pollutants produced during sample processing and analytical preparation rather than physiologically circulating particles. A more biologically relevant estimate of systemic load is provided by mass-based measurement (µg/mL). Small sample sizes and inadequately characterized exposure variables, such as nutrition, drinking water, inhalation, and socioeconomic status, continue to limit current research [4,16].

Recent experimental evidence suggests that chitosan derived from *Procambarus clarkii* (PCC) can form structured networks in gastric conditions capable of entrapping MPs and promoting their fecal elimination [17]. The trapping mechanism occurs in the stomach, where chitosan becomes fully solubilized and forms micro-networks stabilized by starches and organic acids such as tartaric acid (added to the formulation) and ascorbic acid (present in foods). Electron microscopy and stereomicroscopy document the formation of these networks, which can incorporate lipid-based substances (i.e., fatty acids, cholesterol) and interact with MPs through ionic bonds and additional forces (i.e., Van der Waals, hydrophobic interactions), generating complex matrices within the chyme [17]. As gastric contents progress into the duodenum and ileum, the rising pH induces gelification of these networks, which maintain their affinity for MPs and prevent contact with enterocytes. Bile salts are co-adsorbed onto these floccules [18], further stabilizing the aggregates and promoting their progression toward the colon rather than translocation across the intestinal epithelium. In the colon, bacterial chitosanases [19,20], partially degrade the chitosan matrix and utilize its saccharide content for trophic purposes -a process described as “shifting” [21,22,23,24]-while the embedded MPs, which lack trophic value for colonic bacteria, are ultimately eliminated with the fecal mass.

Within this framework, the use of PCC represents a rational and timely strategy to investigate whether targeted gastrointestinal sequestration can modulate circulating MPs levels in humans. Due to low-micron particles persisting in blood due to incomplete phagocytic clearance and limited renal elimination, an intervention capable of enhancing intestinal sequestration and prevention of absorption could provide the first controlled evidence of internal MPs load dynamics. Assessing particle kinetics in apparently healthy volunteers before and after a defined period of PCC administration offers a unique opportunity to determine whether systemic MPs burden is modifiable and to establish foundational parameters for future clinical and environmental health studies. Quantification using mass (µg/mL) yields a more reliable and biologically significant estimate of systemic MP load. intestinal sequestration and prevention of absorption

The aims of the present pilot study were to: (i) quantify baseline MP concentrations in human blood using mass-based metrics; (ii) evaluate changes following a 15-day PCC intervention; and (iii) explore any potential relationships between MP levels and individual variables (i.e., age, sex, body mass index (BMI), and medical-environmental exposure). Additionally, a control group was included to provide environmental exposure and temporal variability into account. To our knowledge, this study provides preliminary evidence of a reduction in circulating MP levels following PCC dietary supplementation and represents an initial attempt to estimate the mean residence time (MRT) of MPs in human blood using a kinetic approach.

## 2. Materials and Methods

### 2.1. Experiment Blueprint

Volunteers were chosen based on inclusion criteria (healthy persons free of shellfish or chitin derivative sensitivities) and exclusion criteria (absence of chronic disease and no known sensitivity to chitosan or shellfish derivatives). All participants provided written informed consent prior to inclusion. The participant also had all of their rights, including the freedom to withdraw at any time without facing consequences. To protect participant data, all PCC administration and stool collection procedures were conducted in accordance with the General Data Protection Regulations (GDPR). The trials have been approved by the International Agency for Pharma Standard Supplements (IAPS) in Pescara, Italy. The code of ethics of experiments that the scientific committee has listed on the approval date of 16 September 2024 is the following: Code MNP-A1 (Figure S4).

**Figure 1:**
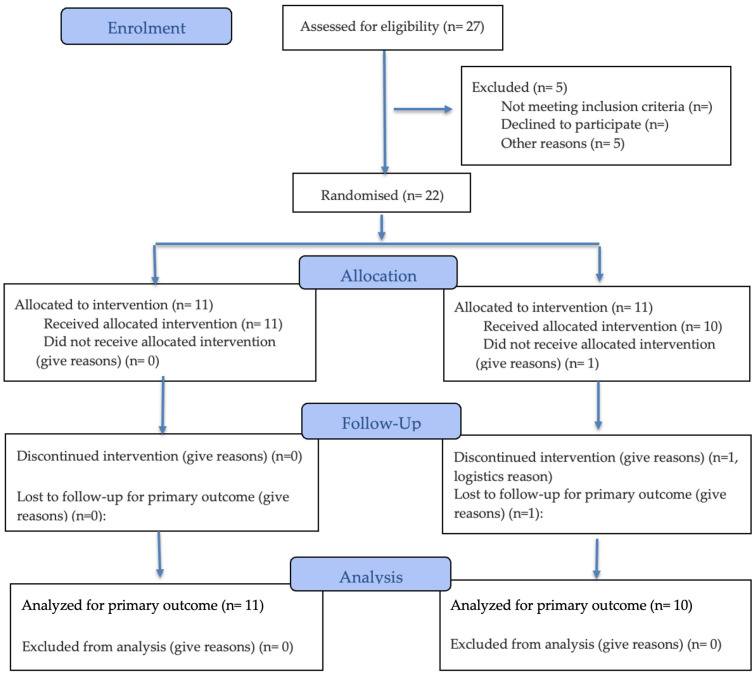
Flow Diagram of the present study (CONSORT 2025).

A 1:1 ratio was employed to assign participants to either the PCC or placebo groups. Instead of using a rigorous basic randomisation approach, a matched assignment technique based on age, sex, and general lifestyle factors has been employed to minimise baseline variability. Throughout the duration of the study, participants and investigators were blinded to group assignment. Due to logistical issues, one participant of the whole recruited cohort was eliminated before the experiment began (Figure 1).

**Figure 2:**
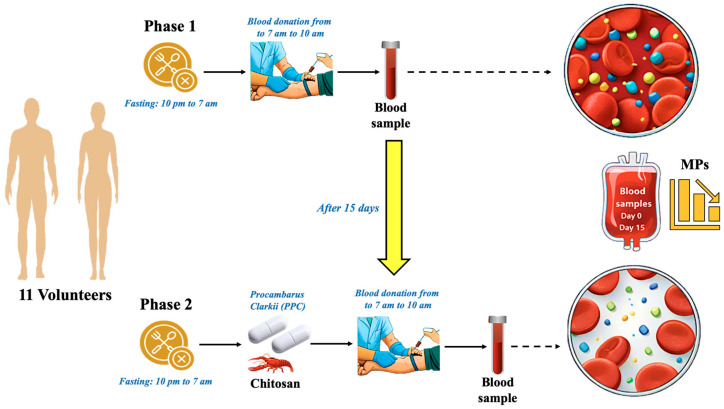
Scheme of the experiment blueprint.

Participants were instructed to maintain their usual diet and lifestyle throughout the study. For 15 days, the intervention group received two capsules containing 0.8 g/day of PCC (0.8 g daily dose of PCC was established using physiological simulations, previous studies, and in vivo and in vitro binding data with MPs) [17,25,26]. The control group received a placebo under identical conditions. Blood samples were collected on day 0 (baseline, Phase 1) and after 15 days (post-intervention, Phase 2) (Figure 2).

A dosage of 0.8 g of PCC was selected in order to mix with the stomach contents and reach a concentration of roughly 1 mg/mL. This quantity was considered to guarantee adequate viscosity and polymer load to efficiently capture the MPs without putting undue strain on the digestive system. 0.8 g of PCC was an appropriate quantity considering that the average volume of human stomach contents following a typical meal is about 800 mL (it might range between 600 and 1000 mL depending on the individual and the type of food). To create a suitably dense gel rich in −NH_3_^+^ sites that are prepared to interact with MPs, about 1 mg of PCC/mL of stomach juice is required. The PCC would stay overly fluid at lower dosages (<0.5 mg/mL), with few accessible −NH_3_^+^ groups close to the MP surfaces. Risks related with greater doses (>2 mg/mL) include excessive viscosity, a discernible delay of gastrointestinal transit, and possible discomfort during swallowing or food release [17].

Therefore, in terms of safety and bioavailability, 0.8 g of PCC is within the tolerance limits for food consumption (up to 3 g/day without creating major side effects). In addition to optimizing MP trapping efficacy, the PCC at this concentration also forms a stable hydrogel at an acidic pH (−NH_3_^+^), allowing for relatively quick transit and removal within 12 h, as required by the protocol. The PCC was food-grade chitosan derived from Procambarus clarkii that was already available as a dietary supplement in Italy [17,24]. With a degree of deacetylation (DDA) of 90%, its inherent viscosity, as measured by Huggins and Kraemer charts [27], varied between 90 and 120 cP (centipoise). The Mark-Houwink-Sakurada algorithm indicated that the PCC polymers had an approximate molecular weight between 100 and 140 kDa. Proton nuclear magnetic resonance (^1^H-NMR) spectroscopy was used to examine the chemical structure of the PCC at 25 °C. Spectra were captured in D_2_O (Figures S1 and S2, Table S1).

After a single daily dose of 0.8 g PCC (for 15 days) and the placebo were provided in two 0.4 g capsules with half a glass of mineral water, two blood samples were taken: one for baseline values (Phase 1) and the other for Phase 2 fifteen days later. Eleven individuals participated in the two experimental stages, as indicated in Figure 2. In parallel, the 10 healthy volunteers assigned to the control group received an equivalent daily dose of 0.8 g of placebo, likewise administered as two 0.4 g capsules with half a glass of mineral water, and blood samples were collected following the same schedule in Phase 1 and Phase 2. Blood samples were obtained after an overnight fast, between 7 and 10 a.m.

Non-invasive testing was necessary and the medication was simply administered once per evening. Payment of any type was requested or given. The IAPS approved the experiment despite PCC actually falling under the food-grade product category [21,24].

### 2.2. Gathering Samples

The Faculty of Chemistry at the University of Oviedo (Spain) performed SEM, µFTIR, and stereomicroscope analysis between January and February 2026, while the Department of Chemistry at the University of Pavia (Italy) performed pretreatment analysis on blood samples and µFTIR analysis. During the screening phase, a thorough evaluation of self-reported medical histories and structured clinical interviews was carried out to determine potential hypersensitivity to chitosan or shellfish. To guarantee safety while preserving the feasibility of the recruiting procedure, participants were eliminated based on documented or self-reported allergic predispositions rather than undergoing formal allergy testing. All participants had to satisfy these requirements in order to be included in the present study.

The study involved eleven otherwise healthy volunteers (five female and six male), aged between 18 and 53 years old, all of whom reported no history of smoking, alcohol consumption, or drug use (Table 1). Participants were required to undergo a fasting period of eight hours prior to blood collection, which was performed in the morning. Table 1 summarizes the general characteristics of these eleven healthy volunteers. Additionally, a control group consisting of ten healthy volunteers was included, comprising five male and five female participants, aged between 20 and 51 years. In accordance with the main study cohort, individuals in the control group also reported no history of smoking, alcohol consumption, or pharmacological treatments (Table 2).

Glass syringes and heparinised tubes were used to collect a 4 mL blood sample with the aim of minimising contamination. For conventional clinical analyses (i.e., blood glucose, cholesterol, liver function, ALT, bilirubin, and kidney function-creatinine), more samples were collected (8 mL of blood). Table S2 summarizes the overall clinical data for the cohort study and Table S3 details the values associated with the control group (placebo). For quality assurance, four heparinized test tubes were maintained at the same temperature as the biological samples (−20 °C) to serve as a blank control. These were subsequently evaluated to account for any potential MP contamination originating from the collection vessels themselves. The clinical and biochemical characteristics of the 11 study participants and 10 volunteers of the control group across Phase 1 (baseline) and Phase 2 are detailed in Tables S2 and S3.

### 2.3. Blood Samples Pretreatment

All of the reagents and distilled water were filtered using a glass micro-fibre filter (0.7 µm particle size, Whatman, Florham Park, NJ, USA) to avoid MP contamination. Each blood sample was placed in a beaker. 100 mL of bi-filtered distilled water was added to the samples to help with stirring. Samples were shaken for 30 min at 120 rpm in a flocculation tester (JLT6, VELPS Scientifica, Usmate Velata, MB, Italy). It has six glass beaker settings, an electrical speed regulator (10–300 rpm), a digital display, and a programmable timer (0–99 h). After that, each blood sample was put in 10 mL of a 50% H_2_O_2_ solution (VWR Chemicals, Briare, France) and left for 24 h at room temperature. After that, 10 mL of Fenton reagent was added to ensure that all organic molecules would oxidize for 24 h at room temperature. Additionally, the material was filtered using a stainless-steel module with overlapping 500, 250, 100, and 20 µm sieves (CISA Sieving Technologies, Barcelona, Spain). After being cleaned, the MPs that were caught on each sieve were gathered in beakers with distilled water that had been filtered twice. The MPs were then separated from inorganic impurities using a density separation approach using a ZnCl_2_ solution (d = 1.5 g/mL, 97% purity, VWR Chemicals, Briare, France). Vacuum glass micro-fibre filters (0.7 µm pore, Whatman, Florham Park, NJ, USA) were used to filter the MP solution.

### 2.4. Microplastic Analysis

A high-resolution color digital camera (Leica DFC310FX; 1.4 Mpixel, CCD, Leica Microsystems CMS GmbH, Wetzlar, Germany) and a semi-automatic stereomicroscope (Leica M205FA, Leica Microsystems CMS GmbH, Wetzlar, Germany) were used for calculating the MPs in the filters. The Confocal UniOvi ImageJ software (Version 1.54p) was also used to estimate the MP-fragment and MP-fibre sizes. The morphology of the MPs discovered in the blood samples (in each Phase) was evaluated using scanning electron microscopy (SEM) analysis (JEOL-6610LV with microanalysis, Tokyo, Japan). The SEM had tungsten filament electron guns, a 0.5–30 kV operational voltage range, and a maximum resolution of 3.0 nm. There was a 5× to 50,000× magnification range. It employed low-vacuum modes for wet or non-conductive surfaces and high-vacuum modes for samples with the best resolution. Backscattered secondary (composition, topography, and shadowing) electron detectors were employed. In addition to the eucentric tilt and rotation, it had a 5-axis asynchronous mechanical eucentric stage that could handle samples as large as 20 cm in diameter. The BMP, TIFF, or JPG image formats were automatically stored by the fully automated SEM, which ran on a single PC.

The chemical composition of the MP was ascertained using a µ-FTIR spectrophotometer (Perkin Elmer Spotlight 200i FTIR spectrophotometer, Springfield, IL, USA) from the Autonomous University of Madrid (UAM) Molecular Spectroscopy Unit. MPs were placed on infrared-light-transparent supports (KBr pellets) for transmission analysis. A spectrum database, which is kept in the aforementioned device and contains over 36,000 spectra of various chemicals, was compared with the outcomes of an automatic analysis of the generated infrared spectra. The measurement parameters utilized for analyzing the MPs were spectral range (550–4000 cm^−1^), resolution (16 cm^−1^), number of scans (30), and infrared beam aperture (20 × 100 microns for MP fibres and 50 × 50 microns for MP-fragments). The results were compared using a spectral database that contained the spectrophotometer of over 36,000 chemicals, including paints and derivatives, polymers, fibres, organic and inorganic materials, solvents, drugs, and more.

A Euromex Edu Blue (v2.4.9.0) magnifying lens (Duiven, The Netherlands) with 20× and 40× magnification was used to examine the micrometric samples once the researcher had determined the sample count for each plate as well as the approximate size, shape, and colour of each plate (data acquired prior to analysis). After being visually identified, the samples were physically placed on the surface of a KBr pellet that had been created. This pellet was transparent in the mid-infrared range, and it was then placed in a specific sample holder within the infrared microscope. For this crucial operation, a magnifying lens was always used as a visual aid. Following that, the micrometric samples were handled using absorbent paper tips or tungsten needle tips, which are primarily used in dentistry and are most suited for micro-fibres with an electrostatic charge. The technician’s expertise was crucial in this phase of the pre-measurement procedure. After each plate sample was placed on the surface of the KBr pellet, the sample container was transferred to the infrared microscopy stage, where each micrometric sample was inspected under a microscope. After the analytical procedure was set up with the aforementioned measurement parameters, the micrometric samples were examined using infrared.

The MPs are identified by comparing the collected spectra with the reference spectra stored in the Spectrum database of the measurement instrument. Falcon tubes and plastic bottles used for sampling and sample handling may leak some MPs. Following a triple analysis, the average MP concentration produced by plastic bottles was 0.71 ± 0.31 MPs/L. Less than 0.5% of the total MP concentration measurable in each experiment is made up of these amounts, which are regarded as insignificant [28,29].

### 2.5. Quality Assurance and Quality Control (QA/QC)

According to a prior study [17], quality control and assurance (QA/QC) were conducted from the time the MP samples were collected until they were quantified. Using glass micro-fibre filters (0.7 µm pore size), eliminating polymeric contaminants in the lab, and filtering chemical reagents before use were all crucial QA/QC procedures. Throughout the sampling and analysis procedure, good field and laboratory procedures (GLPs) were employed to reduce secondary contamination from MPs that were discovered in the air, on surfaces, and ultimately on the equipment. Thus, the least amount of plastic was used in the creation of the sampling and processing samples. Procedural blanks were used where this was not possible. It was thought that using Falcon tubes and plastic bottles for sample handling and sampling might release a certain number of MPs. To avoid any interference with the research, control trials were carried out in both scenarios. Falcon tubes and plastic bottles released MP at mean concentrations of 0.4 ± 0.1 MPs/L and 0.3 ± 0.1 MPs/L, respectively. Their contamination was deemed minor because they made up less than 0.5% of the total MP concentration employed in each experiment [17,28]. Every experiment and analysis was carried out in triplicate.

To prevent sample contamination, all blood sample filtration procedures were performed in a certified laminar flow cabinet under strict observation. Due to its unidirectional flow of sterile, particle-free air, this type of cabinet provides a clean-air working environment that satisfies ISO 5 criteria. MP contamination management techniques frequently incorporate this kind of equipment.

### 2.6. Statistics

Due to the study being primarily exploratory and heuristic in its nature, statistical analyses were carried out to find initial trends rather than to prove causal correlations. All comparisons were handled as paired observations since the same information about individuals was collected both before (Phase 1) and after (Phase 2) the intervention. A heuristic, exploratory approach was used to determine the sample size. Considering the paired structure and the expected directionality of change, the sample size of at least 11 cases was deemed sufficient to offer preliminary proof of the modifiability of systemic MPs burden after 15 days of PCC administration. The Wilcoxon signed-rank test was used in the primary analysis to compare MP concentrations before and after treatment. The mean ± SD will be used to report descriptive statistics. All calculations were performed using JMP. Statistical significance was set at *p* < 0.05, by SAS analyses (version 9.4; SAS Institute Inc., Cary, NC, USA).

## 3. Results

### 3.1. Comparative MP Analysis Between Phase 1 (Baseline) and Phase 2 (PCC) in Cohort Study

Table 3 shows the MP concentrations in the blood samples of each volunteer, before and after PCC administration, along with the percentage reduction in blood MP levels. The use of PCC resulted in a 26.3 ± 10.1% decrease in MP concentration (Mann–Whitney U test, *p* < 0.05).

The control group, which consisted of ten participants, remained unchanged throughout the duration of the study (Table 4). There was no statistically significant difference between the mean MP concentration at Phase 1 (1.87 ± 0.28 μg/mL) and Phase 2 (1.85 ± 0.30 μg/mL), exhibiting a 3.0% mean variation. Additionally, the MP concentration (63 ± 9 MPs/10 mL vs. 64 ± 11 MPs/10 mL) remained almost unaltered. These findings demonstrate that endogenous MP levels do not spontaneously decrease under controlled conditions in the absence of PCC intervention (Table 4).

**Figure 3:**
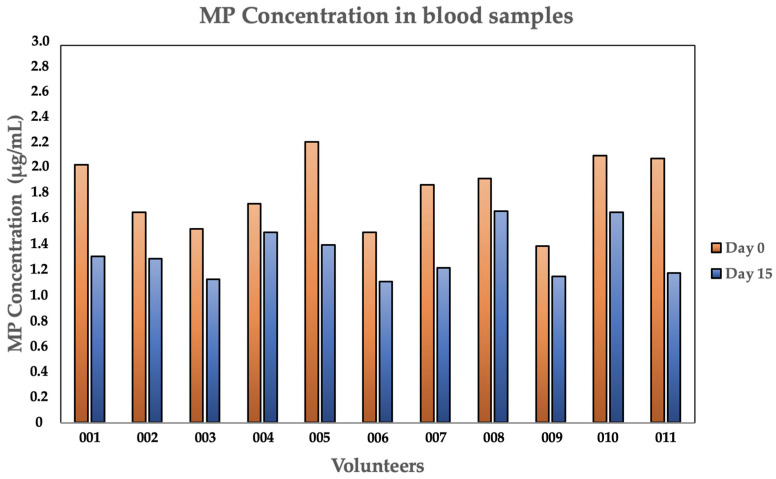
MP concentrations of each volunteer, in Phase 1 vs. Phase 2, in the cohort study.

Figure S5 exhibits examples of MPs found and analyzed in blood samples. Figure 3 exhibits the MP concentrations in both phases of the cohort study (Phase 1 and Phase 2).

**Figure 4:**
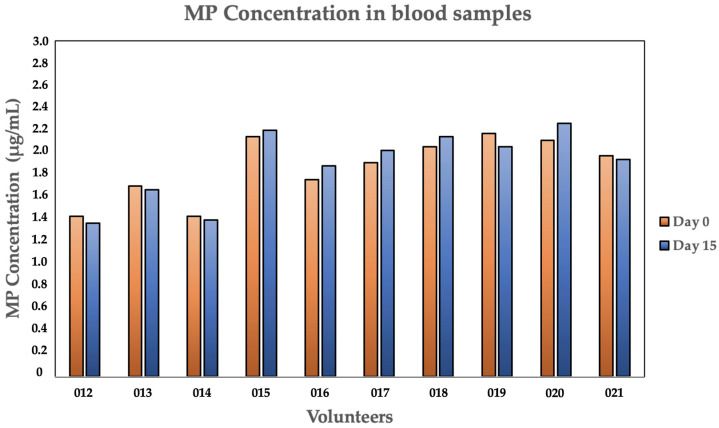
MP concentrations of each volunteer, in Phase 1 vs. Phase 2, in the control group.

On the other hand, an arbitrary distribution of MP concentrations during the same period of time is presented in Figure 4 (Control Group). The placebo group exhibited very little variance, in contrast to the cohort study: Across Phase 1 and Phase 2, the majority of volunteers (i.e., 012, 013, 014, and 021) maintained concentrations that were almost equal. Significant increases in MP levels were observed in Volunteers 015, 016, 017, 018, and 020. This is probably due to ongoing exposure to the environment and diet in the absence of an active clearance agent.

**Figure 5:**
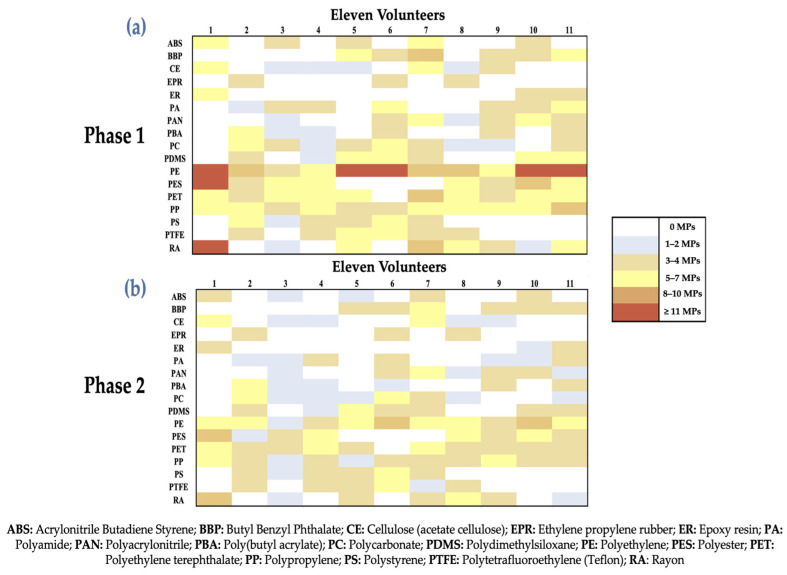
Heatmap of each volunteer; (**a**) Phase 1 (baseline), (**b**) Phase 2 (after PCC administration), in the cohort study.

Considering that the control group either remained statistically and visually stable or slightly increased over the same 14-day period (Figure 5), the comparison of these findings provides supportive evidence that the reduction observed in the treatment group (Figure 4) is consistent with an effect of PCC administration and not a result of natural physiological variation or a controlled diet.

Using Micro-Fourier Transform Infrared Spectroscopy analysis (µFTIR), blood samples from this study revealed 17 different types of MPs, both before (Phase 1) and after administration of 0.8 g of PCC (Phase 2) (Figure S3a,b). The following MPs were analysed: Acrylonitrile butadiene styrene (ABS), butyl benzyl phthalate (BBP), cellulose (CE, acetate cellulose), ethylene propylen rubber (EPR), epoxy resin (ER), polyamide (PA), polyacrylonitrile (PAN), poly(butyl acrylate (PBA), polycarbonate (PC), polydimethylsiloxane (PDMS), polyethylene (PE), polyester (PES), polyethylene terephthalate (PET), polypropylene (PP), polystyrene (PS), polytetrafluoroethylene / teflon (PTFE), and rayon (RA). With considerable variations depending on the MP chemical composition, statistical analysis generally indicated a significant decrease in blood MP concentrations after PCC, both unconditionally and substantially. Future research should increase the sample size, widen the sampling window, and use multiple comparison settings to validate these findings [3,30,31].

In the cohort study, all size fractions indicated a decrease in the total quantity of circulating MPs after administering oral PCC (0.8 g/day for 15 days in a cycle) in comparison to the baseline levels. For the 0–10 µm fraction, mean MP counts decreased from 18 ± 5 to 15 ± 5; for the 11–30 µm fraction, from 22 ± 8 to 14 ± 4; for the 31–50 µm fraction, from 10 ± 5 to 6 ± 3; and for the 51–80 µm fraction, from 5 ± 2 to 4 ± 1. The relative size distribution appeared to be increasing simultaneously. After treatment, the percentage of MPs in the 0–10 µm range rose from 35 ± 11% to 38 ± 9%, while the fraction in the 11–30 µm range fell from 39 ± 6% to 35 ± 10%. The 51–80 µm and 31–50 µm fractions’ respective percentage contributions remained fairly stable at 17 ± 6% vs. 16 ± 6% and 9 ± 4% vs. 10 ± 3%. These findings indicate that after PCC treatment, the MP concentration decreased globally and was redistributed toward smaller particle sizes. The percentages of MPs analysed according to size ranges (Phase 1 and Phase 2) are presented in Table S4 (Cohort study) and in Table S5 (Control group).

In all blood samples, circulating MPs have been identified to include 17 different types of polymers. PE (11/11, 100%), PP (11/11, 100%), PET (10/11, 91%), PDMS (7/11, 64%), PC (9/11, 82%), PA (7/11, 64%), and PAN (7/11, 64%) were the most common polymers at baseline (Phase 1). ABS (5/11, 45%), BBP (6/11, 55%), CE (7/11, 64%), ER (3/11, 27%), EPR (3/11, 27%), PS (6/11, 55%), PTFE (6/11, 55%), and RA (8/11, 73%) were less common polymers. When oral PCC (0.8 g/day) was administered for 15 days, the total MP values decreased from 600 to 472 (−21.3%). While the qualitative polymer profile was essentially unchanged, reductions were particularly noticeable for PE, PP, PET, and PDMS. The 11–30 µm and 31–50 µm fractions, which contained a majority of PE, PP, and PET-derived particles, showed significant decreases when integrated with size-class data. The 0–10 µm percentage rose proportionately (from 35 ± 11% to 38 ± 9%) but declined slightly in absolute terms. The proportions between 31 and 50 µm and 51 and 80 µm remained constant (17 ± 6% vs. 16 ± 6% and 9 ± 4% vs. 10 ± 3%). A substantial decrease in total MPs following treatment was confirmed by Wilcoxon testing (*p* < 0.01). Table S4 provides a summary of all these outcomes. Table S5 provides a summary of all the findings about the control group.

All the healthy volunteers reported no adverse effects after receiving PCC.

The irregular and angular MPs in Figure S6, which consist of fragments of about 57 µm and 65 µm, are consistent with the secondary fragmentation of larger polymers. One MP, measuring around 31 µm, had a distinctly spherical shape, which is typical of microbeads used in personal hygiene products and some industrial uses. The surfaces of the particles varied from smooth to porous and deteriorated, and some had scaly textures and tiny crevices. Small MPs (1.9–5.0 µm) and larger fragments (>60 µm) were included in the size distribution. Angular fragments ranging in size from 15 to 65 µm exhibited asymmetric geometries, surface ruptures, and uneven edges. The size range of 1.9–5 µm was the most common for granular and ellipsoidal MPs. Several MPs had rough, porous surfaces, which suggested a greater surface area. These areas implied that the MPs and the nearby biological material were closely related. In the circulatory compartment, larger particles up to around 60 µm in size have been identified.

The polymeric landscape’s granular analysis (Tables S6 and S7) indicated an eclectic composition of 16 MP types, with PE and PP predominant. All identified species, including technical resins like ABS and PTFE, demonstrated a unidirectional downward trend in the PCC group (cohort study), whereas these MP concentrations persisted or even slightly rose in the control group, indicating ongoing environmental exposure in the absence of a clearance agent.

### 3.2. MP Concentrations in Blood and MRT

Table 5 reports the MPs in Phase 1 and Phase 2 blood levels. The Mean Residence Time (*MRT*) related to the MP pool’s residence time may be determined by combining the values of the two points (Phase 1 vs. Phase 2) and the following algorithm:

$$MRT=\frac{15}{\ln\left(\frac{t_{15}}{t_{0}}\right)}$$

where:

15 = number of days of treatment;
*t*_0_ = Concentration of MPs in phase 1 (µg/mL);
*t*_15_ = Concentration of MPs in phase 2 (µg/mL).

**Table 5:** Comparison of MP blood levels between Phase 1 and Phase 2 (Mean ± SD), including percentage decrease and MRT, in the cohort study.

Healthy Volunteer	MP Concentration (µg/mL)		% Decrease in MP Concentrations	MRT (Days)
	Phase 1	Phase 2		
001	2.05	1.32	35.6	34.1
002	1.67	1.30	22.2	59.6
003	1.54	1.14	26.0	49.7
004	1.74	1.51	13.2	106.4
005	2.23	1.41	36.8	32.8
006	1.51	1.12	25.8	50.3
007	1.89	1.23	34.9	35.0
008	1.94	1.68	13.4	105.0
009	1.40	1.16	17.1	79.4
010	2.21	1.67	21.2	62.8
011	2.10	1.19	43.3	26.5
MEAN	1.84	1.34 *	26.3	58.3
SD	0.28	0.20	10.1	28.0

* Statistical significance was determined using Tukey’s post hoc test; *p* < 0.01 indicates a significant difference between Phase 1 and Phase 2.

MP blood levels decreased by 26% (Mann–Whitney; *p* < 0.01; Cohen 0.999). The subjects’ age (r = −0.291; *p* > 0.05) and body weight (r = 0.291; *p* > 0.05) did not demonstrate any Spearman correlation. Additionally, 58 ± 28 days was the MRT for the entire cohort; nevertheless, there was significant inter-individual heterogeneity, with the range being 27 to 106 days.

The MRT was calculated only for the polymer detected in all 11 samples, a criterion met exclusively by patients with PE and PP. Their respective MRTs were 46 ± 28 days and 33 ± 11 days. These estimates reflect the intrinsic residence time of each polymer more correctly and exhibit considerably higher internal consistency, even if they are lower than the values obtained from the entire polymer mixture.

### 3.3. Heatmap and Boxplots Analysis

**Figure 6:**
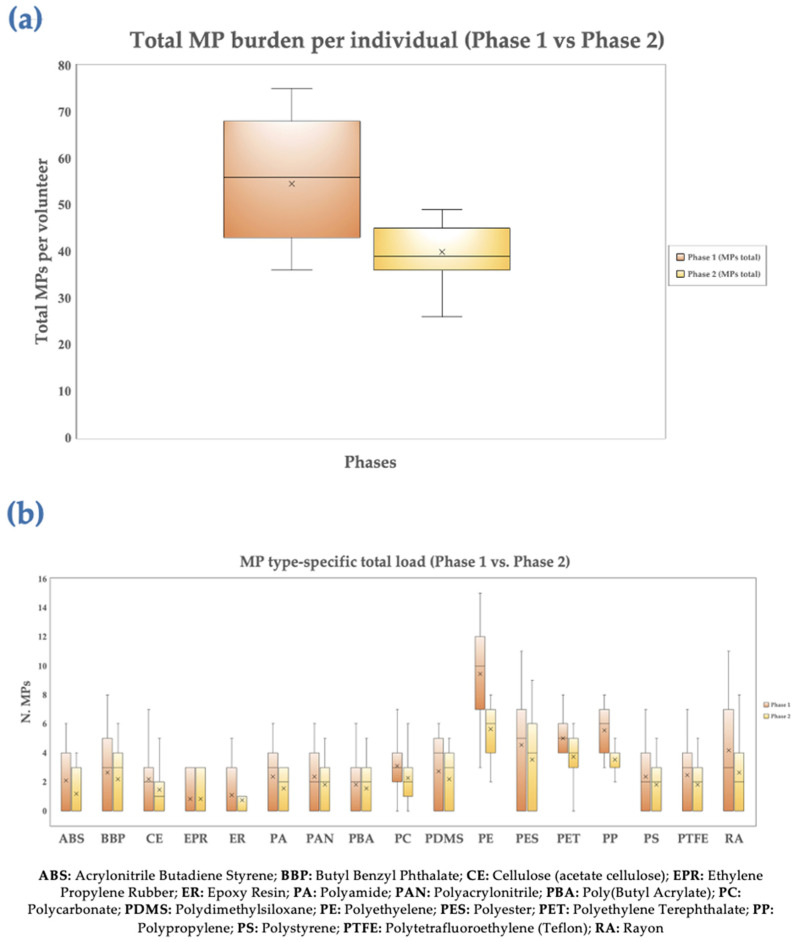
Boxplots of paired comparison of total and polymer-resolved circulating MP concentration; (**a**) Phase 1 (baseline) and (**b**) Phase 2 (after PCC administration), in the cohort study.

A heatmap is included to help visualize the data gathered in both phases and aid in identifying any possible relationships between each of the MPs (Figure 5). Boxplot representations were created in order to identify outliers and examine the distributions of the different MPs in the eleven healthy volunteers in both periods (Figure 6).

Eleven healthy volunteers were evaluated in two phases: Phase 1 (baseline), in which peripheral blood samples were collected on the morning of day zero while fasting, and Phase 2 (PCC), in which peripheral blood samples were obtained on the morning of day 15 after receiving 0.8 g of PCC daily for 15 days. A semi-quantitative abundance scale was employed to demonstrate the results, with 0 MPs (white), 1–2 MPs (blue), 3–5 MPs (yellow), 8–10 MPs (orange), and ≥11 MPs (red) representing the various MPs. During Phase 1, MPs with a complex and varied polymeric pattern were widely found in all volunteers. PE, PET, and PP were the most common MPs, with high values (8–10 and ≥11 MPs) in a number of volunteers, especially volunteers 1, 5, 6, 10, and 11. MPs with significant interindividual variability, including PES, PC, and PDMS, were identified at intermediate concentrations. Furthermore, other MPs (such as ABS, CE, ER, and PTFE) exhibited irregular frequency or low levels, indicating less reliable exposure sources.

This pattern demonstrates long-term exposure to MPs in the environment, with polyolefins and polyesters clearly dominating, which is in line with their extensive use and high levels of industrial production (Figure 5a). After 15 days of PCC administration, most volunteers in Phase 2 exhibited a general decrease in chromatic intensity in different MPs. For PET, PP, PES, PC, and PDMS, this decrease was very significant. Despite being the most common MPs, PE observed a relative decrease in several groups of volunteers (i.e., volunteers 1, 5, 6, and 10), shifting from the ≥11 MPs group to the 5–7 or 8–10 MPs ranges. MPs remained in all individuals, suggesting that the intervention moderated rather than entirely removed the systemic burden (Figure 5b).

The distribution of the total MPs burden identified in peripheral blood samples collected from 11 fasting healthy volunteers prior to (Phase 1) and after (Phase 2) a 15-day PCC (0.8 g/day) treatment is illustrated in Figure 6a. As a heuristic exploratory study, this study was not intended to test a predetermined causal theory, but rather to detect initial tendencies. The overall MP burden in Phase 1 ranged roughly from 35 to 75 MPs per participant, with a comparatively high median value and significant inter-individual heterogeneity. The significant variation in baseline MP exposure and/or accumulation among individuals is demonstrated by the large interquartile range. The distribution exhibited an evident downward shift after the intervention (Phase 2). Both the total range and the interquartile range shrank, and the median MP burden decreased to about 40 MPs per volunteer. Similar trends occurred in the mean values (shown by ×), indicating that the reduction in data is not due to the influence of single outliers but rather to a generalized decrease throughout the cohort.

Overall, the data demonstrate that after 15 days of PCC supplementation, there was a roughly 25–30% relative decrease in total circulating MPs. The same individuals were evaluated before and after the intervention; therefore, paired statistical testing was used to compare the total circulating MPs burden between Phases 1 and 2. The Shapiro–Wilk test was used to determine whether the paired differences were normal. Phase 1 and Phase 2 values were compared using a paired Student’s *t*-test once the assumption of normalcy was met. The non-parametric Wilcoxon signed-rank test was used when normality was not established. Cohen’s d for parametric analysis or rank-biserial correlation for non-parametric analysis was used to determine the effect size. The contrast shown in Figure 5a is consistent with this analysis.

The MP load distributions for both phases, stratified by polymer type, are illustrated in Figure 6b. Compared to other polymers, including PTFE, EPR, and ER, PE, PET, and PP showed the highest median values in Phase 1, suggesting their dominance in circulating MPs. The majority of polymer classes exhibited a generalized decrease in median and mean values following PCC supplementation (Phase 2). PE, PET, and PP showed the biggest decreases, and their interquartile ranges similarly contracted. Polymers such as ABS, CE, PA, PC, and PDMS indicated moderate decreases, although significant inter-individual variability persisted. Polymers such as EPR, ER, and PTFE, on the contrary, indicated relatively few modifications and a significant overlap between Phase 1 and Phase 2 distributions. All of these results indicate a polymer-dependent pattern of decrease after the intervention, indicating that particular MP types are more or less susceptible to removal via PCC. Heterogeneous responses to the intervention were found using polymer-resolved paired analysis. Following Benjamini–Hochberg false discovery rate (FDR) correction, substantial or almost significant decreases in PE, PET, and PP were observed with effect sizes falling between modest and moderate. While EPR, ER, and PTFE revealed minimal to no phase-to-phase variation, other polymers, such as ABS, CE, PA, PC, and PDMS, displayed non-significant decreasing tendencies. These results suggest that PCC’s impact on circulating MPs varies depending on the polymer, with stronger statistical evidence supporting a decrease in the most prevalent and hydrophobic polymer classes.

## 4. Discussion

This pilot study investigated the variation in blood levels of MPs in eleven healthy volunteers who were employed in the healthcare sector and resided in industrialized metropolitan regions after receiving a PCC orally for 15 days in consecutive days. The cohort’s geographic and occupational homogeneity permits a study of the influence of common sources of exposure, both occupational and environmental, and minimizes the variability related to wide environmental variances. Polymers, including PAN, PET, PES, PP, PE, EPR, PDMS, PC, and PTFE, are frequently discovered in the identified polymer profile [29,32,33]. This trend implies a combination of urban environmental sources and, most importantly, occupational sources rather than just conventional dietary exposure.

This pilot study provides initial evidence that gastrointestinal sequestration using PCC may reduce circulating MP levels in humans. The observed reduction is consistent with a mechanism involving decreased intestinal absorption rather than enhanced systemic clearance. The therapeutic potential of PCC-based treatments to decrease systemic MP accumulation is highlighted by the significant distinction between the PCC-treated cohort and the placebo group. The PCC group demonstrated a significant sequestration effect that tended to persist regardless of the initial MP load or polymer chemistry, whereas the control group demonstrated the persistence of common synthetic polymers—likely due to the ubiquitous nature of dietary and environmental exposure (Tables S6 and S7). The polycationic structure of PCC could enable interactions with the multiple surface charges (eco-coronas) of circulating MPs, as indicated by the non-selective removal of both highly hydrophobic particles (such as PS and PE) and more polar fragments (i.e., PA and PAN). Despite the broad spectrum of MPs identified, the reduction in mass concentration and total particle count demonstrates that PCC effectively inhibits MPs’ natural bioaccumulation, providing a significant advantage over the random fluctuations observed in the control group.

The possible therapeutic efficacy of PCC is determined by a broad-spectrum sequestration capacity rather than a tight selectivity for specific polymer types, according to a comparative analysis of Tables S6 and S7. Highly non-polar polyolefins (i.e., PE, PP), aromatic polymers (i.e., PS, PET), and technical elastomers (such as EPR and ABS) were within the chemically diverse spectrum of MPs that demonstrated a significant decrease in the cohort study (Table S6). This lack of chemical selectivity suggests that surface adsorption and electrostatic attraction, rather than particular covalent bonds, are probably what control the interaction between PCC and circulating MPs. Once internalized in biological fluids, the majority of MPs develop a “eco-corona”—a layer of lipids and proteins that usually imparts a net negative surface charge. The simultaneous clearing of both fibrous (i.e., RA and PA) and granular (such as PVC and PC) particles may be interpreted by the polycationic nature of the PCC derivative used in this study, which seems to enable a generalized affinity for these negatively charged coronas. The control group (Table S7), on the other hand, lacked any such clearance levels, with certain prevalent polymers, such as PE and PET, maintaining stable or even rising frequencies (i.e., Volunteers 015 and 021). This divergence shows that the PCC intervention provides a strong, broad-spectrum reduction across polymer types, a mechanism that may mitigate the steady-state accumulation of multiple MP types in the human bloodstream, whereas environmental intake is continuous and non-discriminatory, as was observed in the placebo group.

**Figure 7:**
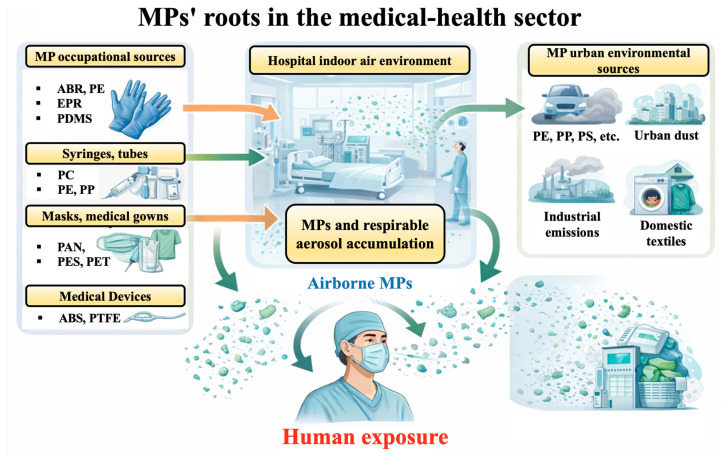
Sources, distribution pathways, and human exposure to airborne MPs in healthcare and urban environments.

Specifically, the presence of EPR, PDMS, PC, and PTFE is consistent with materials used in medical devices, gaskets, gloves, tubing, and parenteral delivery systems, whereas the detection of PAN, PES, PET and RA is consistent with the release of microfragments/microfibres from technical textiles and medical clothing (gowns, scrubs, masks) (Figure 7) [29,32,33].

Other MPs, such as ABS and EPR, are frequently encountered in urban sources such as furniture, shoe bottoms, road traffic (tire wear), and electronic equipment casings. Their identification in every participant suggests a background environmental exposure linked to urban living that coincides with the particular professional exposure in the medical field [34,35]. Furthermore, the identification of PBA, ER, and plasticizers such as BBP indicates the involvement of paints, adhesives, vinyl flooring, and coatings that are present in both home and healthcare environments [36,37]. Due to age and abrasion, these materials may emit MP-fragments, particularly in indoor spaces where human activity is prevalent. When considered collectively, these findings provide credibility to the hypothesis that the hospital setting functions as a microenvironment enhanced with respirable MPs, particularly those of medical equipment and technical textiles. The coexistence of industrial and urban polymers suggests long-term mixed exposure, which raises significant concerns regarding the long-term effects of systemic MP accumulation in healthcare professionals.

Inhalation seemed to be the most prevalent form of exposure. The mechanical wear of polymers, recirculated airflow, thorough cleaning procedures, and the constant handling of plastic items all contribute to the high concentration of airborne particles found in hospital settings. Several studies have shown that inhaled micro-nanoparticles can deposit in the alveolar epithelium and then pass through the alveolar-capillary barrier and enter the systemic circulation as MPs or NPs [7]. For polymers such as PAN, PET, and PES, which are commonly found in hospital interior air, this process is especially plausible [38,39].

The most significant finding of the present study is the identification of MPs in blood at easily quantifiable levels in individuals who exhibited no signs of persistent illness in the lab. Participants who were significantly underweight or overweight did not exhibit abnormalities in coagulation, cardiovascular, hepatic, renal, or haematological parameters other than the presence of MPs. This finding emphasizes an important issue in MP toxicology: MP exposure currently lacks a defined clinical phenotype, and the exposure amounts and durations necessary to cause overt pathology are still unclear, despite mounting evidence of biological interaction. The estimated MRT of circulating MPs is the topic of an additional remarkable finding. The MRT was roughly 58 days for all polymers; more reliable values of ~33 days for PE and ~46 days for PP were obtained when analysis was limited to polymers that were consistently found in all samples. This is the first effort that we have knowledge of to measure these pharmacokinetic characteristics in humans.

A helpful analogy for the present scenario of MP research is provided by Clarke’s description of vitamin E as “a vitamin looking for a disease” [40]. Despite the growing number of exposure evaluations and mechanistic evidence, a particular pathological endpoint associated with MPs has not yet been identified. According to experimental data, MPs can cause oxidative stress and inflammatory reactions by generating reactive oxygen species, disrupting membranes, and triggering inflammatory pathways. Particle size, shape, surface chemistry, and the composition of the adsorbed biocorona all affect how they behave biologically [12]. These processes indicate biological activity, but they do not identify a specific disease entity.

Additionally, MPs act as carriers of flame retardants, plasticizers, and adsorbed environmental pollutants, including metals and polycyclic aromatic hydrocarbons (PAHs) [41,42]. The toxicity of the polymer may be increased by this “Trojan horse” effect. According to this idea, MPs should be considered risk modifiers rather than the main cause of disease; they are unlikely to cause disease on their own, but when co-factors are present, they may change the likelihood of disease onset, severity, and timing [43,44]. Therefore, a conceptual reframing is necessary for the identification of polymers such as PE, PP, and PVC in individuals without obvious medical issues. MPs should be considered to be elements of a recently developed “background” internal environment rather than as indicators of an obvious illness. A baseline contamination state is defined by the presence of MPs in blood, and based on exposure duration, cumulative tissue burden, and interactions with referred to determinants of pathology, such as genetic susceptibility, metabolic dysfunction, infections, and lifestyle factors, disease may or may not develop from this state. Therefore, rather than identifying a particular pathology, MPs alter the biological environment in which disease can arise.

Current healthy carriers may reflect a preclinical or subclinical period of changed risk, and it is conceivable that long-term exposure is necessary for circulating MPs to manifest as clinically evident disease. No particular polymer type identified in this cohort seemed to be responsible for any particular clinical outcomes, and given the individuals’ very young ages, prolonged exposure might be required before overt disease emerges. Thus, MPs might be figuratively characterized as their clinical implications remain unclear, fostering an environment that is pro-inflammatory and pro-oxidant, which may facilitate the emergence of metabolic, cardiovascular, and other chronic illnesses. The convergence of exposure time, tissue accumulation, genetic predisposition, and concomitant diseases probably determines the change from minimal exposure to overt disease.

The “Chicago cluster” [14] is a constellation consisting of approximately thirty clinical and subclinical manifestations that have been identified through a synthesis of the literature. Considering its initial clinical expression and latency are still unclear, it has been suggested that this constellation may develop into an “MP syndrome”. Therefore, longitudinal studies that connect internal MP loads to clinical outcomes are crucial to establishing a causal connection. According to a study on atheromatous plaques [45] and progressive cerebral and hepatic accumulation [46], blood MP-NP levels could be an excellent marker of tissue accumulation and rising tissue concentrations are a risk factor. Blood MP concentrations decreased by 26% after 15 days of PCC treatment. This decrease is presumably due to increased excretion of food-ingested particles in the stools and particles swallowed following respiratory exposure. MP bioavailability is decreased and further accumulation is prevented by chitosan–MP complexes that stick to the mucus layer and are carried into the colon [17]. PCC works primarily within the intestinal lumen through local physicochemical interactions rather than systemic bioavailability because chitosan is not absorbed in polymeric form [25].

### 4.1. Size-Dependent Effects of PCC on Circulating MPs

Particle size has been demonstrated to possess a significant impact on the systemic distribution and clearance of MPs [47,48]. Preferential removal of medium-sized MPs is indicated by a substantial decrease in intermediate-sized particles (11–30 µm and 31–50 µm) and the relative enrichment of the smallest fraction (0–10 µm), whereas smaller particles appear less susceptible to clearance. PCC’s physicochemical characteristics as a polycationic biopolymer with a high adsorption capacity [17] are consistent with this pattern. It may encourage electrostatic binding to negatively charged MP surfaces and facilitate particle aggregation and subsequent removal. Previous studies have reported similar sorption and aggregation behaviors between charged polymers and MP surfaces [49]. A selective interaction compared to a nonspecific reduction across all sizes is further supported by the relative stability of the biggest size fraction. Furthermore, a consistent response to PCC among volunteers is suggested by the reduced interindividual variability observed after treatment, which is compatible with size-dependent clearance processes reported for particulate pollutants [50]. The longitudinal within-subject design and the consistent size-selective shift in MP distribution offer mechanistic evidence for a chitosan-mediated reduction in systemic MP load, despite the fact that the lack of a placebo-controlled group restricts causal inference. These results demonstrate PCC’s potential role as a detoxifying agent that can alter MP size distribution and bioavailability in vivo.

### 4.2. Polymer-Specific Effects of PCC on Circulating MPs

According to the present study, PCC administration reduces circulating MPs in multiple polymer classes rather than one in particular. The prevalence of PE and PP is in accordance with their broad use in consumer items and food packaging, as well as their pervasive occurrence in the environment [48]. PE, PP, PET, and PDMS all demonstrated significant post-treatment decreases, which points to a broad-spectrum impact of PCC on systemic MP load. This response can be understood mechanistically by integrating the polymer composition with the particle size distribution. A size-dependent rather than polymer-specific removal mechanism is supported by the preferential reduction in MPs in intermediate size fractions (11–30 µm and 31–50 µm), which are mainly composed of particles derived from PE, PP, and PET. PCC’s physicochemical characteristics as a polycationic biopolymer with a high adsorption capacity [17] are consistent with this pattern. It may help electrostatically bind to negatively charged or oxidized MP surfaces and encourage aggregation into complexes that are easier to remove from the bloodstream. Since smaller particles are predicted to have a higher translocation potential and a lower adsorption efficiency, the relative enrichment of the smallest size fraction following treatment lends additional credence to this interpretation. The paired longitudinal design and the concordant size- and polymer-dependent effects offer convergent evidence for a PCC-mediated decrease in systemic MP bioavailability, despite the absence of a placebo-controlled group limiting causal inference.

An interesting aspect emerged from the analysis of the apparent distribution volume (Vd), which was calculated for PE as it was the only polymer present in all cases. Based on non-compartmental kinetics, it emerged that the Vd was 0.26 L, an extremely limited value not compatible with blood volume and/or the volume of any organ. The only hypothesis that can be formulated is that Vd represents macrophages, which account for only a limited portion of the tissues. If these data were confirmed by larger studies, it would indicate that the toxicological key of small MPs and/or NPs is linked to the activation of macrophages and not to direct tissue damage

### 4.3. SEM-Based Characterization of the Morphology and Toxicological Relevance of Circulating MPs

The identification of both spherical and irregular/angular MPs suggests that primary particles, including microbeads from industrial and cosmetic applications, and secondary particles produced by polymer degradation coexist. Multiple environmental issues and degradation pathways are implied by this morphological variety. Particles smaller than 10 µm are more likely to pass through epithelial barriers and reach the systemic circulation, rendering the reported size range toxicologically relevant. Their sizes mirror those of granular and ellipsoidal MPs between 1.9 and 5.0 µm, which is particularly concerning, since they may be simpler for macrophages and reticuloendothelial system cells to absorb [4].

A higher specific surface area could enhance the adsorption of heavy metals and persistent organic pollutants from biological fluids, as evidenced by the porous and damaged surfaces of many MPs. This lends credence to the hypothesis that after internalization, MPs could serve as carriers of environmental pollutants. It is anticipated that MPs will quickly establish a biomolecular corona in blood that consists mainly of plasma proteins, including albumin and fibrinogen [12]. This procedure can affect immunological recognition, cellular interactions, and biodistribution. This protein adsorption phenomenon may be reflected in the apparent connection of MPs with filamentous material in micrographs. MPs identified that individuals who seem to be in excellent health are a sign of broad exposure. The bloodstream’s existence of fragments up to 60 µm raises significant concerns about epithelial translocation and possible roles in chronic inflammation, oxidative stress, and the transport of pollutants across the circulatory system [48].

### 4.4. Heatmap and Boxplot Analysis of Polymer-Specific Changes in Circulating MPs Following PCC Intervention

The relative haematic burden of MPs has generally decreased after PCC administration, according to a comparative heatmap analysis between Phase 1 and Phase 2 (Figure 5a,b). This effect was more noticeable for hydrophobic polymers, specifically PET, PP, and PES, indicating that more hydrophobic polymers may be preferentially involved in gastrointestinal tract adsorption activities. Individual differences in intestinal transit and metabolism, ongoing environmental exposure, and PCC’s varying affinity for specific polymers are all implied by the fact that the degree of reduction was not consistent across subjects or polymer types. These results lend credence to the theory that PCC may function as a gastric MP sequestrant (intestinal sequestration and prevention of absorption), hence restricting systemic absorption as opposed to immediately improving systemic elimination [17,51].

Comparable reductions were not seen in all kinds of polymers. In contrast to PS and PTFE, which exhibited erratic responses, PET, PP, PES, PC, and PDMS consistently decreased. Despite a little decline following the intervention, PE remained the most prevalent polymer. According to this differential behavior, PCC reveals selective interactions that are reliant on the physicochemical characteristics of the polymer, such as hydrophobicity, surface charge, and molecular structure, rather than acting as a non-specific scavenger. Mechanistically, this suggests that rather than being controlled by a consistent adsorption mechanism, PCC efficacy is influenced by polymer properties. Phase 2 similarly indicated a decrease in polymeric variety, with fewer polymer types per individual and a decreased representation of high-abundance groups. This reduction in compositional complexity may have toxicological significance since it may lessen concurrent exposure to several additives specific to polymers and pollutants linked to their surfaces. The persistence of MPs following intervention, nevertheless, suggests either sluggish clearance via hepatosplenic or renal pathways, stable systemic biodistribution, or possible tissue reservoirs (Figure 5b) [17,51].

Furthermore, boxplot analyses indicate a consistent decrease in mean and median circulating MP levels after short-term PCC administration, along with a decrease in interindividual dispersion (Figure 6a,b). Intestinal uptake or baseline exposure may be homogenized by the intervention, as this pattern indicates a systematic rather than stochastic effect. The idea of polymer-dependent efficacy is supported by the observed polymer-resolved trends, with larger decreases in PE, PET, and PP perhaps due to a combination of hydrophobic, hydrogen-bonding, and electrostatic interactions between MPs and PCC [17,51]. Due to the exploratory character of the present study, the small sample size, and the lack of a control group, clear causal inference is not possible despite these consistent trends. It is impossible to rule out nutritional or behavioral complicating factors. The concept that PCC might act as a polymer-modulated MP bio-sequestrant is supported by the stable directionality of alterations. These preliminary findings should be validated in more extensive, controlled studies that include kinetic modeling of MP absorption and elimination as well as absolute quantification.

### 4.5. Systemic Distribution of MPs and Their Potential Human Health Risk (i.e., Cardiovascular and Neurovascular Pathology)

Blood, saphenous vein tissue, bone marrow, and other bodily fluids have all been found to contain MPs [52,53]. This indicates that MPs that are inhaled, consumed, or come into contact with the skin can pass through biological barriers and enter the bloodstream.

**Figure 8:**
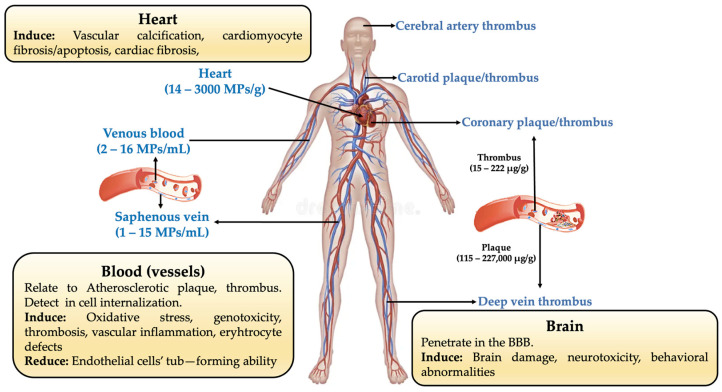
MP distribution and toxicological effect in the circulatory system.

Their continued presence in areas that are typically shielded by physiological membranes points to restricted clearance and potential tissue retention. Crucially, MPs have been found in artery tissues with and without atherosclerotic plaques, with plaque-bearing arteries exhibiting noticeably greater amounts. A possible connection between MP accumulation and atherosclerosis processes is supported by this spatial relationship. Clinical observations serve as more evidence for this concern. Carotid plaques are known to include jagged MPs, which have been linked in long-term follow-up studies to higher risks of myocardial infarction, stroke, and death [52,53]. Although in vitro studies at environmentally realistic levels have generated conflicting results, experimental models offer mechanistic support, demonstrating that long-term exposure to MPs might worsen vascular inflammation and plaque formation. Collectively, these findings indicate MPs and atherosclerosis have a complicated connection that is probably influenced by dose, particle size, and polymer type (Figure 8) [52].

In the erythrocyte membrane, MPs can adsorb, alter the ζ potential, decrease deformability, cause vesiculation (micro-vesicles), and, at high doses, cause hemolysis. This fact is critical since the erythrocytes are very sensitive to mechanical and oxidative stress, lack a nucleus, and are unable to repair their membrane [54]. Furthermore, MPs may raise reactive oxygen species (ROS) levels and play a crucial role in eryptosis activation [54,55]. It is important for consumption into account that MPs may directly enter the bloodstream through infusion, parenteral nutrition, and transfusion systems [51,54,55]. Biliary excretion, reticuloendothelial sequestration (mainly in the liver and spleen), phagocytosis by circulating and tissue-resident macrophages, and minimal renal filtration limited to the smallest NP fractions are the main physiological mechanisms controlling the systemic clearance of MPs and NPs. Nevertheless, hepatic clearance is noticeably sluggish and size-restricted, and phagocytic absorption frequently generates intracellular sequestration rather than true systemic evacuation, suggesting that both mechanisms are inherently ineffective for thorough elimination. Larger MPs also surpass the physiological glomerular filtration limits. When these restrictions are captured into consideration in conjunction with ongoing exposure to the environment, the constant flow of particles probably exceeds the body’s limited ability to eliminate them, which eventually allows for longer residence durations and cumulative tissue persistence.

### 4.6. Comparative Analysis of Internalised MPs: Our Findings vs. Global Literature Benchmarks

MPs are widely internalized throughout human haematological compartments worldwide, according to data collected in Table S8. MPs are widely internalized throughout human haematological compartments worldwide, according to data collected in Table S2. Despite the fact that reported quantities vary by several orders of magnitude, from ng/mL to μg/mL, this heterogeneity is mostly due to systematic differences in extraction procedures and analytical detection limits rather than varying environmental exposures. While the identification of specialized elastomers and biopolymers (i.e., Polylactic acid; PLA) indicates a complex, multifactorial exposure matrix encompassing domestic, textile, and medical sources, a notable convergence in polymer profiles, dominated by PE, PP, and PET, reflects global production trends [56].

Micro-scale fragments and fibres (1–800 μm) predominate morphologically; nevertheless, a major diagnostic gap is still caused by the systematic exclusion of the small MPs and NPs portion due to existing technology limitations. Adopting integrated, standardized analytical frameworks is necessary due to the inherent trade-offs between mass-based quantification, such as Pyrolysis-Gas Chromatography-Mass Spectrometry (Py-GC-MS) and vibrational spectroscopy (μFTIR, micro-Raman spectroscopy; μRaman) [57]. According to this study, healthcare professionals had a median concentration range of 1.40–2.23 μg/mL, suggesting that clinical settings, which are marked by extensive usage of personal protective equipment (PPE) and polymer-based devices, may provide special exposure pathways [57,58,59]. The ability of MPs to pass through the main biological barriers and persist systemically is supported by these observations considered collectively. To properly characterize the toxicological concerns associated with chronic plastic bioaccumulation, it is essential to address existing constraints, such as the absence of standardized quantification units and longitudinal clinical correlations [60,61,62,63,64,65,66,67].

## 5. Putative Limitations of the Study

This preliminary study emphasized the significance of pilot studies and the need for additional research using a greater range of participants. High-resolution μRaman spectroscopy, occasionally referred to as atomic force microscopy–Raman (AFM–Raman), is one complementary technique that may detect particles smaller than 10 µm, allowing for even more precise characterization in subsequent research. This experiment did not represent daily exposure, chronic exposure, dietary variations, or environmental pollutants in the general population because of the meticulously controlled indoor/outdoor environment in which it was carried out [57,58]. Additionally, despite the fact that the focus of this study was gastrointestinal sequestration, we acknowledge that the lack of concurrent urinary monitoring is a limitation. Incorporating such analyses in future studies would be an important complementary endpoint to further clarify the relative contributions of intestinal sequestration versus renal clearance in systemic MP kinetics [68,69,70].

A complementary multimodal analytical approach that uses methods like µ-FTIR, Laser Direct Infrared (LDIR), and µ-Raman to verify polymer identity while maintaining information on particle size and morphology is the most effective way to measure MPs in human blood sample matrices with accuracy. Other mass-based methods that offer polymer-specific charge metrics and are frequently more appropriate for clinical study, such as dual-shot Py-GC/MS and, for specific polymers, liquid chromatography-tandem mass spectrometry-coupled depolymerization (LC-MS/MS), are integrated with this [57].

## 6. Conclusions

In this pilot cohort, oral PCC treatment was associated with a notable reduction in circulating MPs (26%). These findings provide plausibility to the concept that gastric sequestration could be a helpful strategy for controlling internal MP burden. This outcome indicates that the product’s mode of action, which is based on the development of micro-networks in the stomach that are stabilized by organic acids and then gelify in the small intestine, acts effectively to restrict MP absorption in real-world scenarios. The exhibited reduction in blood concentrations, despite the brief duration of the treatment period, indicates that lowering the daily burden of absorbable particles may quickly alter the intake-clearance balance. To validate these findings and assess long-term effects, more comprehensive, controlled studies must be conducted. This pilot study provides preliminary evidence that dietary supplementation with PCC may be a potential strategy warranting further investigation to reduce indoor plastic pollution. According to the data, this might be a useful public health tool in the Plasticene/Anthropocene age rather than just a supplement.

## Figures and Tables

**Table 1:** General characteristics of the eleven volunteers in the cohort study (Mean ± SD).

Volunteers	Sex	Age (Year)	Height (m)	Weight (Kg)	BMI (Kg/m^2^)	Area of Residence	Professional Sector
*001*	M	42	1.82	92	27.8	Metropolitan area	Healthcare sector
*002*	F	43	1.65	52	19.1		
*003*	F	19	1.60	45	17.6		
*004*	F	18	1.70	60	20.8		
*005*	M	46	1.80	110	34.0		
*006*	M	49	1.70	100	34.6		
*007*	M	52	1.75	85	27.8		
*008*	F	48	1.68	76	26.9		
*009*	F	53	1.60	54	21.1		
*010*	M	35	1.62	50	19.1		
*011*	M	25	1.75	63	20.6		
*MEAN*		39	1.70	72	24.5	-	
*SD*		13	0.08	22	6.1	-	

**Table 2:** General characteristics of the ten volunteers in the control group (Mean ± SD).

Volunteers	Sex	Age (Year)	Height (m)	Weight (Kg)	BMI (Kg/m^2^)	Area of Residence	Professional Sector
*012*	F	20	1.64	55	20.4	Metropolitan area	Healthcare sector
*013*	M	43	1.75	81	26.4		
*014*	M	26	1.81	78	23.8		
*015*	M	51	1.74	83	27.4		
*016*	F	35	1.60	62	24.2		
*017*	F	29	1.71	63	21.5		
*018*	F	47	1.62	58	22.1		
*019*	M	32	1.88	80	23.2		
*020*	F	46	1.67	66	23.7		
*021*	M	24	1.84	84	24.8		
*MEAN*		35	1.73	74	24.5	-	
*SD*		11	0.10	12	6.1	-	

**Table 3:** MP concentrations in blood samples in Phase 1 and Phase 2 after PCC treatment in the cohort study (Mean ± SD).

Volunteer	Phase 1 N. MPs/10 mL	Phase 2 N. MPs/10 mL	Phase 1 MP Conc. (µg/mL)	Phase 2 MP Conc. (µg/mL)	MP Removal (%)	MP Type *
001	69	49	2.05	1.31	35.6	ABS, CE, ER, PA, PAN, PE, PES, PET, PP, RA
002	51	40	1.67	1.30	22.2	EPR, PA, PBA, PC, PDMS, PE, PES, PET, PP, PS, PTFE
003	36	26	1.54	1.14	26.0	ABS, CE, PA, PAN, PBA, PC, PE, PES, PET, PP, PS, RA
004	40	36	1.74	1.51	13.2	CE, PA, PBA, PC, PDMS, PE, PES, PET, PP, PS, PTFE
005	57	36	2.23	1.41	36.8	ABS, BBP, CE, PC, PDMS, PE, PET, PP, PS, PTFE, RA
006	61	45	1.51	1.12	25.8	BBP, EPR, PA, PAN, PBA, PC, PDMS, PE, PP, PS, PTFE
007	75	49	1.89	1.23	34.9	ABS, BBP, CE, PAN, PC, PDMS, PE, PET, PP, PS, PTFE, RA
008	44	37	1.94	1.68	13.4	CE, EPR, PAN, PC, PE, PES, PET, PP, PTFE, RA
009	43	38	1.40	1.16	17.1	BBP, CE, PA, PAN, PBA, PC, PE, PES, PET, PP, RA
010	56	44	2.12	1.67	21.2	ABS, BBP, ER, PA, PAN, PDMS, PE, PES, PET, PP, RA
011	68	39	2.10	1.19	43.3	BBP, ER, PA, PAN, PBA, PC, PDMS, PE, PES, PET, PP, RA
MEAN	55	40	1.84	1.34	26.3	-
SD	13	7	0.28	0.20	10.1	-

* ABS: Acrylonitrile Butadiene Styrene; BBP: Butyl Benzyl Phthalate; CE: Cellulose (acetate cellulose); EPR: Ethylene propylene rubber; ER: Epoxy resin; PA: Polyamide; PAN: Polyacrylonitrile; PBA: Poly(butyl acrylate); PC: Polycarbonate; PDMS: Polydimethylsiloxane; PE: Polyethylene; PES: Polyester; PET: Polyethylene terephthalate; PP: Polypropylene; PS: Polystyrene; PTFE: Polytetrafluoroethylene (Teflon); RA: Rayon.

**Table 4:** MP concentrations in blood samples in Phase 1 and Phase 2 in the control group (Mean ± SD).

Volunteer	Phase 1 N. MPs/10 mL	Phase 2 N. MPs/10 mL	Phase 1 MP Conc. (µg/mL)	Phase 2 MP Conc. (µg/mL)	MP Removal (%)	MP Type *
012	48	46	1.43	1.37	4.2	ABS, CE, EPR, ER, PA, PE, PES, PET, RA
013	57	56	1.70	1.67	1.8	BBP, CE, PA, PBA, PC, PE, PES, PET, PP, PS
014	48	47	1.43	1.40	2.1	CE, PA, PAN, PBA, PC, PE, PES, PET, PP, PS
015	72	74	2.14	2.20	-	ABS, EPR, PA, PBA, PC, PDMS, PE, PET, PP, PS, PTFE, RA
016	59	63	1.76	1.88	-	ABS, BBP, CE, EPR, PDMS, PE, PET, PP, PTFE, RA
017	64	68	1.91	2.02	-	BBP, EPR, PA, PAN, PBA, PC, PDMS, PE, PES, PP, PS, PTFE
018	69	72	2.05	2.14	-	ABS, BBP, CE, PC, PDMS, PE, PET, PP, PS, PTFE, RA
019	73	69	2.17	2.05	5,5	ABS, BBP, CE, EPR, PAN, PC, PE, PES, PET, PP, PTFE, RA
020	71	76	2.11	2.26	-	PA, PAN, PBA, PC, PDMS PE, PES, PET, PP, PS, RA
021	66	65	1.97	1.94	1.5	ABS, EPR, PA, PBA, PC, PDMS, PE, PET, PP, RA
MEAN	63	64	1.87	1.85	3.0	-
SD	9	11	0.28	0.30	1.8	-

* ABS: Acrylonitrile Butadiene Styrene; BBP: Butyl Benzyl Phthalate; CE: Cellulose (acetate cellulose); EPR: Ethylene propylene rubber; ER: Epoxy resin; PA: Polyamide; PAN: Polyacrylonitrile; PBA: Poly(butyl acrylate); PC: Polycarbonate; PDMS: Polydimethylsiloxane; PE: Polyethylene; PES: Polyester; PET: Polyethylene terephthalate; PP: Polypropylene; PS: Polystyrene; PTFE: Polytetrafluoroethylene (Teflon); RA: Rayon.

## Data Availability

The original contributions presented in this study are included in the article/Supplementary Material. Further inquiries can be directed to the corresponding author.

## Supplementary Materials

The following supporting information can be downloaded at https://www.mdpi.com/article/10.3390/jox16030092/s1: Figure S1: Technical data sheet of PCC; Figure S2: ^1^H-NMR spectra of PCC recorded in D_2_O at 25 °C; Figure S3a: µFTIR spectra of MPs identified in the present pilot study (cohort study and control group); Figure S3b: µFTIR spectra of MPs identified in the present pilot study (continued); Figure S4: Ethical Committee Approval; Figure S5: Stereomicroscope images of MPs identified in this study: from (a–c) MPs in Phase 1 samples; from (d–f) MPs in Phase 2 samples (in cohort study and control group); Figure S6: Illustrative SEM images of MPs identified in the blood samples, in the present study; Table S1: ^1^H-NMR spectral parameters of PCC; Table S2: Baseline clinical and biochemical characteristics of the cohort study (Mean ± SD); Table S3: Baseline clinical and biochemical characteristics of the control group (Mean ± SD); Table S4: Distribution of MP size classes (count and percentage) by the eleven volunteers involved in the cohort study (Mean ± SD); Table S5: Distribution of MP size classes (count and percentage) by the ten volunteers involved in the cohort study (Mean ± SD); Table S6: Type and frequency of MPs in each volunteer involved in the cohort study; Table S7: Type and frequency of MPs in each volunteer involved in the control group; Table S8: Studies on MPs in human blood in the literature; Written informed consent model.

## Abbreviations

The following abbreviations are used in this manuscript:

ABS	Acrylonitrile Butadiene Styrene
AFM-Raman	Atomic Force Microscopy-Raman
BBB	Blood–Brain Barrier
BBP	Butyl Benzyl Phthalate
BMI	Body Mass Index
CE	Cellulose (cellulose acetate)
CNS	Central Nervous System
cP	Centipoise
CSF	Cerebrospinal Fluid
DDA	Degree of Deacetylation
EPR	Ethylene Propylene Rubber
ER	Epoxy Resin
FDR	Benjamini–Hochberg False Discovery Rate
GDPR	General Data Protection Regulations
GLPs	Good Field and Laboratory Procedures
GUT	Gastrointestinal Tract
^1^H-NMR	Proton Nuclear Magnetic Resonance
IAPS	International Agency for Pharma Standard Supplements
LC-MS/MS	Liquid Chromatography-tandem Mass Spectrometry
LDIR	Laser Direct Infrared
MPs	Microplastics
MRT	Mean Residence Time
NPs	Nanoplastics
PA	Polyamide
PAHs	Polycyclic Aromatic Hydrocarbons
PAN	Polyacrlonitrile
PBA	Polu(butyl acrylate)
PC	Polycarbonate
PCC	*Procambarus clarkii* Chitosan
PDMS	Polydimethylsiloxane
PE	Polyethylene
PES	Polyester
PET	Polyethylene Terephthalate
PLA	Polylactic Acid
PP	Polypropylene
PPE	Personal Protective Equipment
PS	Polystyrene
PTFE	Polytetrafluoroethylene
Py-GC-MS	Pyrolysis–Gas Chromatography-Mass Spectrometry
RA	Rayon
ROS	Reactive Oxygen Species
SEM	Scanning Electron Microscopy
Vd	Distribution Volume
µFTIR	Micro-Fourier Transform Infrared Spectroscopy
µRaman	Micro-Raman Spectroscopy
