# Supplementary material for: Reduction in Circulating Microplastics in Humans Following Gastrointestinal Sequestration by Chitosan: A Pilot Controlled Study
Source: J Xenobiot. 2026 May 22;16(3):92. doi: 10.3390/jox16030092 (PMC13214702; doi:10.3390/jox16030092)

Journal of *Xenobiotics*

MDPI

Supplementary Materials: Reduction in Circulating Microplastics in Humans Following Gastrointestinal Sequestration by Chitosan: A Pilot Controlled Study

Umberto Cornelli, Giovanni Belcaro and Claudio Casella

Figure S1. Technical data sheet of PCC.

Nutratrade

YOUR TRUST IS OUR SUCCESS®

NUTRATRADE S.r.l.		Certificate of analysis	
Name **Chitosan**		SOURCE: **PROCAMBARUS CLARKII**	
Issued by	*QA*	Approved by	*Riccardo Cultraro*
Manufacturing date: **31/08/2025**	Best Before: **30/08/2027**	Batch number: **2025083114**	

**Product Name**	Chitosan	**Solvent Used**	NA
**Country Origin**	China	**Grade**	Food ☒ Feed ☐ Pharma ☐
**CAS No.**	9012-76-4	**Quantity**	300kg
**Part No.**	4.010-126.0	**Shelf life**	24 months when properly stored
**Molecular Formula**	$C_{56}H_{103}N_9O_{39}$		
**Packing**	Paper-drums and two plastic-bags inside, food contact material compliant with EC Reg. 1935/2004 and EU Reg. 10/2011	**Storage**	Store in cool & dry place. Do not freeze. Keep away from strong light and heat.
**Analysis Item**	**Specification**	**Method**	**Result**
Deacetylated Degree	＞90%	NA	90.27%
DER	NA	NA	NA

**Chemical and Physical Characteristics**			
**Analysis Item**	**Standard value**	**Method**	**Result**
Appearance	Powder	Visual	Conform
Color	White to light yellow	Visual	Conform
Odor& Taste	Characteristic	Organoleptic	Conform
Identification	Positive	NA	Conform
Excipient	NA	NA	NA
Loss on drying	≤10.0%	Ph.Eur.9.0<2.2.32>	6.17%
Total ash	< 1%	Ph.Eur.9.0<2.4.16>	0.23%
Solubility in Water	NA	Organoleptic	NA
Solubility in oil	NA	NA	NA
Apparent density	NA	Eur.Ph.<2.9.34>	NA
Tapped density	NA	NA	NA
Solubility((in 1% Acetic Acid))	> 99.0%	NA	99.86%
Viscosity	90-130 mPa·s(cP)	NA	Conform
Sieve analysis	95% through 100 mesh	USP39<786>	Conform
Arsenic (As)	NMT 1 ppm- Reg.EU 2023/915	Ph.Eur.9.0<2.2.58>ICP-MS	Conform
Cadmium (Cd)	NMT 1 ppm- Reg.EU 2023/915	Ph.Eur.9.0<2.2.58>ICP-MS	Conform
Lead (Pb)	NMT 3 ppm- Reg.EU 2023/915	Ph.Eur.9.0<2.2.58>ICP-MS	Conform
Mercury (Hg)	NMT 0.1 ppm - Reg.EU 2023/915	Ph.Eur.9.0<2.2.58>ICP-MS	Conform
Heavy Metals	NMT 10 ppm- Reg.EU 2023/915	Ph.Eur.9.0 <2.4.8>	Conform
Pesticides Residues	Conform Reg.(EC) .396/2005 and amendments.	Gas Chromatography	Conform
Solvent Residues	Conform Eur.Ph. 9.0 <5,4 > and EC Dir. 2009/32	Ph.Eur.9.0<2.4.24>	Conform
Hydrocarbons PAHs	≤ 50 ppb -Reg.EU 2023/915	GC-MS	Conform
Benzo(a)pyrene	≤ 10 ppb -Reg.EU 2023/915	GC-MS	Conform
Radioactivity	≤ 600 Bq/Kg - Reg. EC 2020/1158	NA	Conform
Aflatoxin B1	≤ 5 ppb - Reg.EU 2023/915	Ph.Eur.9.0<2.8.18>	Conform
Aflatoxins Σ B1, B2, G1, G2	≤ 10 ppb - Reg.EU 2023/915	Ph.Eur.9.0<2.8.18>	Conform

NUTRATRADE S.R.L. | Via Como, 57/B, 20033 SOLARO (MI) | P.IVA 08547400963 | capitale sociale € 1.000.000,00 interamente versato

+39 02.83.96.17.59 info@nutratrade.it www.nutratradesrl.com

Figure S2. ^{1}H-NMR spectra of PCC recorded in D_2O at 25 °C.

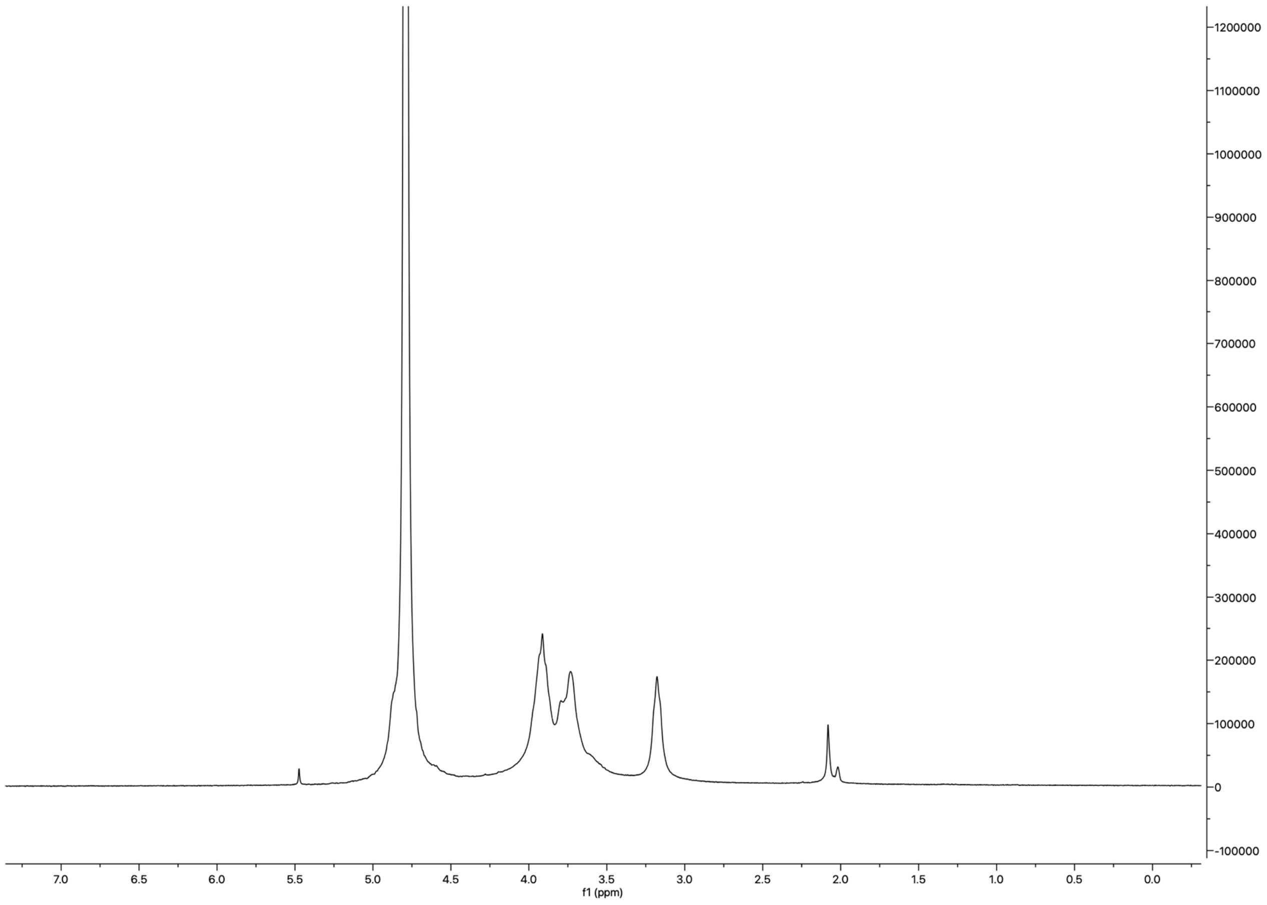

Table S1. ^{1}H-NMR spectral parameters of PCC

Parameters	Results
H-1 peak	masked by HOD* solvent signal.
3–4 ppm region	Present
CH_3CO (~2 ppm)	Weak
Line shape	Slightly broader
Noise	Low

**HOD: Hydrogen-Oxygen-Deuterium*

The noticeably reduced strength of the methyl proton (CH_3CO) signal at about 2.0 ppm indicates a high DDA (DDA > 90%) in the PCC sample, which is confirmed by the ^{1}H-NMR spectra (Figure S2). The experimental spectrum's modest resonance is in line with the acetyl groups' almost total elimination during processing. The distinctive resonances of the glucosamine backbone are visible in the PCC ^{1}H NMR spectra. The H_2 to H_2 protons of the pyranose ring are responsible for the multiplet signals observed in the 3.2–4.1 ppm range. The methyl protons of the N-acetyl group ($-NHCOCH_3$) correlate to a residual signal at about 2.0 ppm, which indicates that the polymer chains have not been fully deacetylated. The strong residual HOD peak, which frequently appears in aqueous-phase NMR spectra of polysaccharides, effectively masks the anomeric signal of the H^{-1} proton (~4.8 ppm) since D_2O is used as a solvent. The signal is still visible in the proton ring region (3.0–4.0 ppm), but the shape seems a little broader. The inherent viscosity of

https://doi.org/10.3390/jox16030092

the acidified solution and the macromolecular structure of chitosan, with its high DDA concentration, are probably the reasons of this minor spectrum widening and resolution. The high signal-to-noise ratio and characteristic chemical shifts confirm both the structural integrity and the high purity of the PCC. Furthermore, the absence of significant signals in the aliphatic region (below 1.5 ppm) indicates a lack of low molecular weight organic contaminants, further validating the sample's purity profile

https://doi.org/10.3390/jox16030092

Table S2. Baseline clinical and biochemical characteristics of the cohort study (Mean ± SD)

Healthy Volunteer	Phase 1 (baseline)											
	Bp Max (mm Hg)	Bp Min (mm Hg)	Hr (bpm)	RBC (10^5)	WBC (10^3)	Hb (g/dL)	ALT (mU/mL)	Bilirubin (mg/dL)	Creatinine (mg/dL)	Total Ch (mg/dL)	Glucose (mg/dL)	TG (mg/mL)
001	127	70	72	5.0	7.5	15.8	19	0.4	1.2	139	92	120
002	135	65	68	4.4	7.1	13.2	12	0.5	0.7	160	95	150
003	140	60	71	4.6	7.2	14.1	14	0.7	0.8	170	100	165
004	125	60	80	5.3	7.5	12.5	15	0.8	1.0	200	85	128
005	139	70	73	4.8	8.1	15.0	21	1.0	0.7	210	86	173
006	130	70	75	5.2	7.9	16.0	9	0.9	1.1	185	79	149
007	145	55	73	4.9	8.1	14.3	13	0.4	0.9	140	95	148
008	140	60	70	4.9	7.3	13.7	18	0.8	0.7	125	93	126
009	130	55	77	5.1	7.7	15.1	16	0.7	0.9	178	101	132
010	140	60	68	5.3	8.4	15.0	16	0.6	1.1	184	90	135
011	125	65	72	5.1	6.4	16.0	7	0.6	0.9	165	89	135
**MEAN**	**134**	**63**	**73**	**5.0**	**7.6**	**14.6**	**15**	**0.7**	**0.9**	**169**	**91**	**142**
**SD**	**7**	**6**	**4**	**0.3**	**0.6**	**1.2**	**4**	**0.2**	**0.2**	**26**	**7**	**17**

Volunteer	Phase 2 (after 15 days)											
	Bp Max (mm Hg)	Bp Min (mm Hg)	Hr (bpm)	RBC (10^5)	WBC (10^3)	Hb (g/dL)	ALT (mU/mL)	Bilirubin (mg/dL)	Creatinine (mg/dL)	Total Ch (mg/dL)	Glucose (mg/dL)	TG (mg/mL)
001	131	72	73	5.1	7.5	13.5	19	0.4	1.1	147	94	116
002	135	65	70	4.5	7.2	13.1	12	0.6	0.7	165	90	150
003	130	60	71	4.7	7.3	14.1	13	0.6	0.8	180	95	145
004	135	60	78	5.2	7.4	12.6	14	0.8	0.9	190	80	129
005	130	70	73	4.9	8.2	15.5	14	1.0	0.7	202	88	135
006	130	70	75	5.1	7.5	16.0	9	0.9	1.1	170	85	180

https://doi.org/10.3390/jox16030092

Table S2. *Continued*

007	140	55	71	5.0	8.1	14.5	13	0.5	0.9	160	92	122
008	140	55	69	5.0	7.2	14.4	19	0.3	0.7	50	98	118
009	130	55	75	5.1	7.7	15.2	16	0.7	0.9	168	92	140
010	135	65	70	5.3	8.3	14.5	17	0.5	1.1	175	94	150
011	135	60	70	5.0	6.3	16.0	8	0.7	0.9	160	92	140
MEAN	134	62	72	5.0	7.5	14.5	14	0.6	0.9	161	91	139
SD	4	6	3	0.2	0.6	1.1	4	0.2	0.2	40	5	18
Tukey test (HSD)	*0.826*	*0.753*	*0.459*	*0.168*	*0.426*	*0.629*	*0,.441*	*0.492*	*0.168*	*0.810*	*0.772*	*0.588*

Table S3. Baseline clinical and biochemical characteristics of the control group (Mean ± SD)

**Healthy Volunteer**	**Phase 1 (baseline)**											
	Bp Max (mm Hg)	Bp Min (mm Hg)	Hr (bpm)	RBC (10^5)	WBC (10^3)	Hb (g/dL)	ALT (mU/mL)	Bilirubin (mg/dL)	Creatinine (mg/dL)	Total Ch (mg/dL)	Glucose (mg/dL)	TG (mg/mL)
012	120	68	64	4.1	7.0	13.2	11	0.4	0.7	134	78	118
013	113	70	74	5.1	7.2	14.1	15	0.8	0.8	158	101	151
014	129	65	71	4.6	6.5	16.0	20	0.6	1.1	164	84	137
015	121	71	69	5.0	7.5	14.9	16	0.7	1.1	180	93	156
016	136	64	80	4.4	8.4	13.7	17	1.0	0.6	114	86	133
017	131	71	72	5.1	8.1	13.8	8	0.6	0.9	131	84	128
018	126	61	77	5.1	7.3	15.4	11	0.7	0.9	156	104	147
019	117	65	69	5.3	8.1	14.6	17	1.0	1.1	129	90	135
020	134	67	79	4.4	7.6	13.3	15	0.7	1.0	143	111	159
021	121	69	71	5.5	8.1	13.4	14	0.8	0.8	126	89	127
**MEAN**	**134**	**63**	**73**	**5.0**	**7.6**	**14.6**	**15**	**0.7**	**0.9**	**169**	**91**	**142**
**SD**	**7**	**6**	**4**	**0.3**	**0.6**	**1.2**	**4**	**0.2**	**0.2**	**26**	**7**	**17**

Volunteer	Phase 2 (after 15 days)											
	Bp Max (mm Hg)	Bp Min (mm Hg)	Hr (bpm)	RBC (10^5)	WBC (10^3)	Hb (g/dL)	ALT (mU/mL)	Bilirubin (mg/dL)	Creatinine (mg/dL)	Total Ch (mg/dL)	Glucose (mg/dL)	TG (mg/mL)
012	125	65	68	4.2	7.3	13.1	12	0.5	0.6	138	84	114
013	118	68	71	5.0	7.0	14.4	14	0.6	0.9	161	96	149
014	131	63	70	4.6	6.6	15.7	17	0.8	0.6	149	92	141
015	119	74	68	5.1	7.4	16.9	19	1.0	1.1	177	88	151
016	124	68	77	4.5	8.1	14.4	14	0.9	0.8	121	92	141
017	135	67	67	5.2	7.7	12.6	11	0.8	1.0	128	93	121
018	124	62	69	5.0	7.7	15.2	14	0.9	0.8	150	101	143
**Table S3.** *Continued*												
019	121	65	70	5.3	8.4	15.6	15	0.7	1.0	118	94	131
020	134	71	73	4.4	7.9	14.9	13	0.6	1.2	141	104	152
021	122	71	72	5.5	8.2	14.2	19	0.6	0.9	131	97	119
**MEAN**	**134**	**62**	**72**	**5.0**	**7.5**	**14.5**	**14**	**0.6**	**0.9**	**161**	**91**	**139**
**SD**	**4**	**6**	**3**	**0.2**	**0.6**	**1.1**	**4**	**0.2**	**0.2**	**40**	**5**	**18**
Tukey test (HSD)	*0.138*	*0.755*	*0.101*	*0.443*	*0.905*	*0.163*	*0,.678*	*0.885*	*0.882*	*0.377*	*0.322*	*0.106*

Figure S3a. μFTIR spectra of MPs identified in the present pilot study (cohort study and control group)

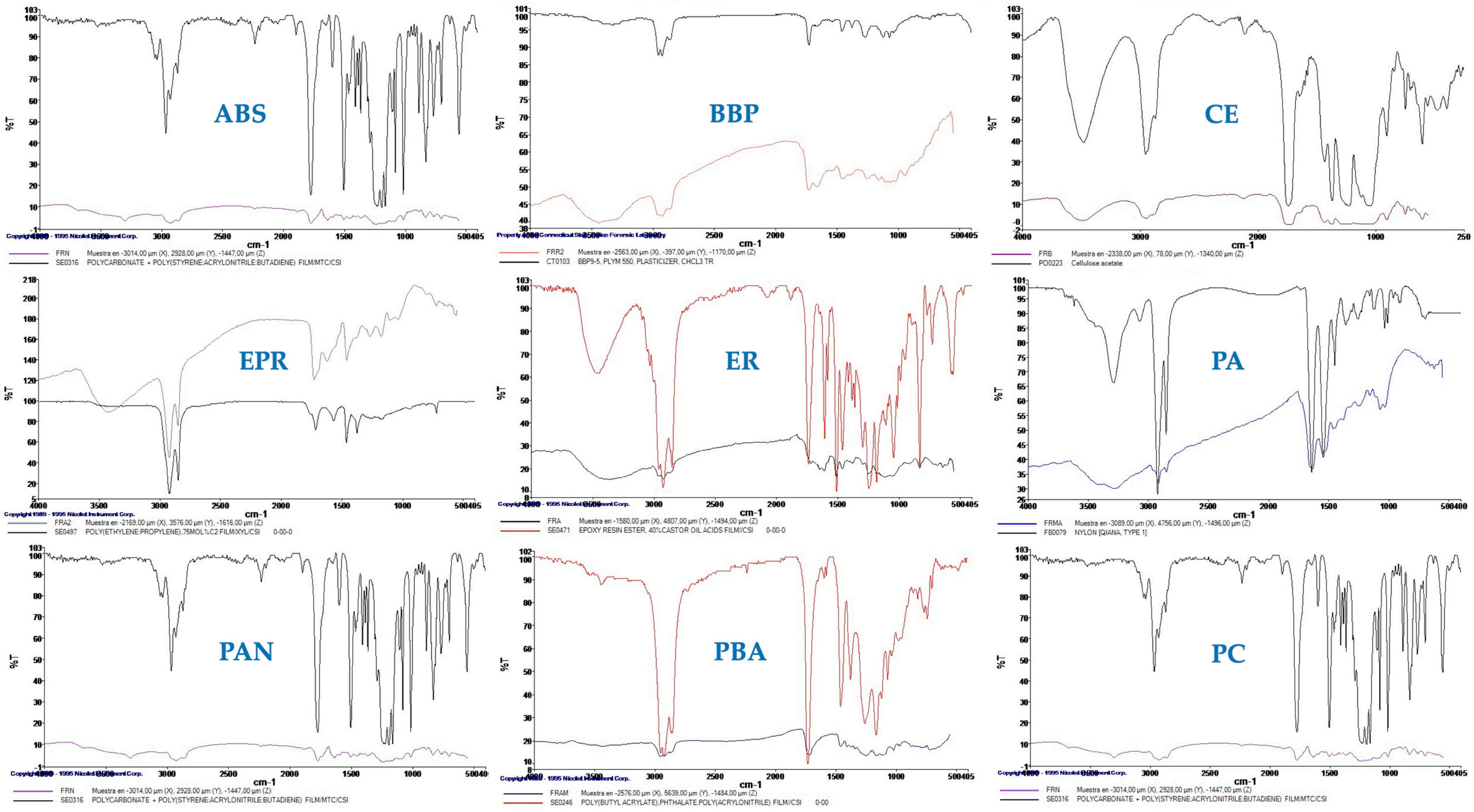

https://doi.org/10.3390/jox16030092

Figure S3b. μFTIR images of MPs identified in the present study (continued)

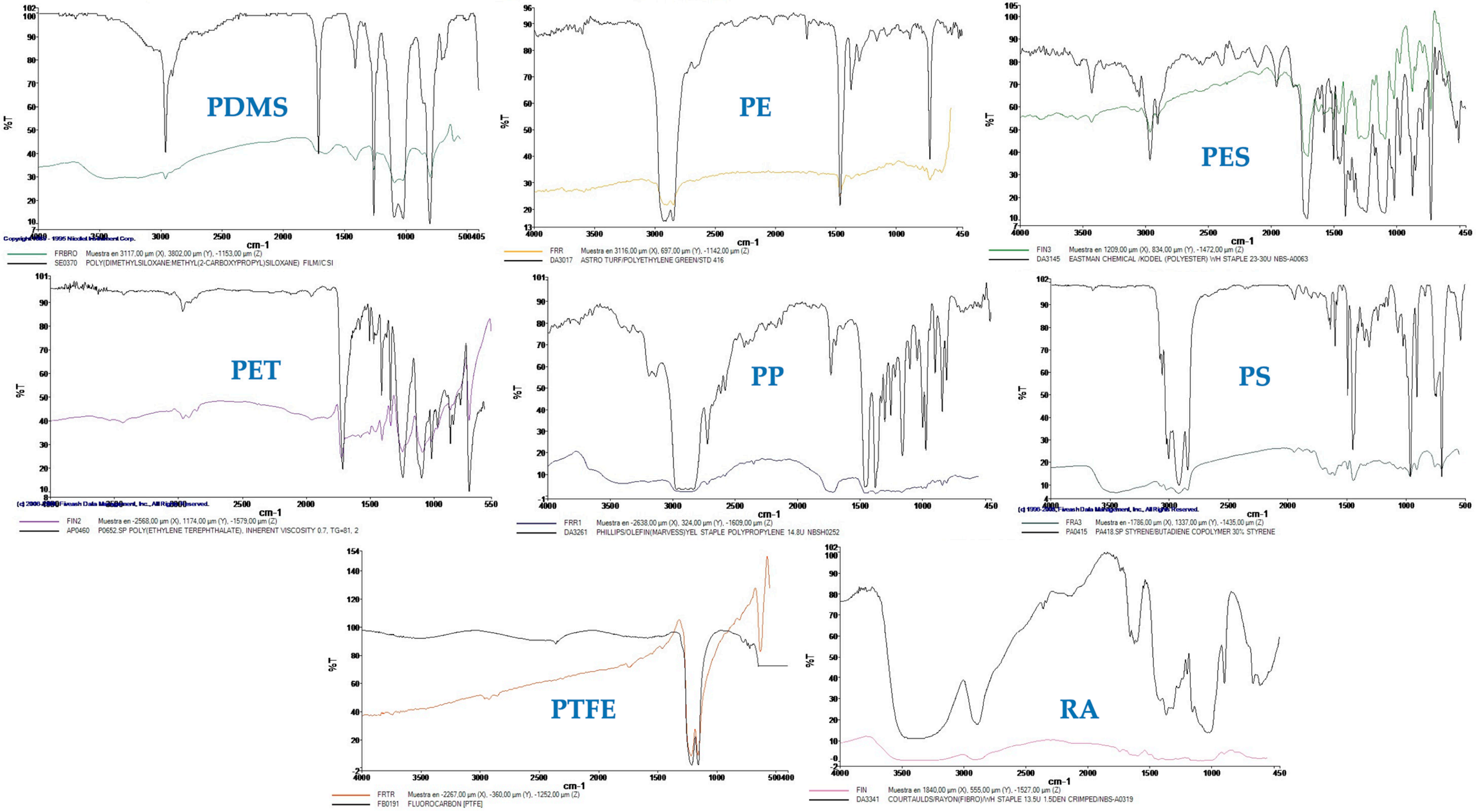

https://doi.org/10.3390/jox16030092

Table S4. Distribution of MP size classes (count and percentage) by the eleven volunteers involved in the cohort study (Mean ± SD)

Healt Volunt	Phase 1								Phase 2							
	N. MP size (μm)				% MP size (μm)				N. MP size (μm)				% MP size (μm)			
	0 - 10	11 - 30	31 - 50	51 - 80	0 - 10	11 - 30	31 - 50	51 - 80	0 - 10	11 - 30	31 - 50	51 - 80	0 - 10	11 - 30	31 - 50	51 - 80
001	16	28	18	7	23	41	26	10	20	13	12	4	41	27	24	8
002	27	19	3	2	53	37	6	4	23	9	5	3	58	23	13	8
003	10	14	6	6	28	39	17	17	11	7	6	2	42	27	23	8
004	18	12	8	2	45	30	20	5	8	21	4	3	22	58	11	8
005	19	24	8	6	33	42	14	11	13	11	7	5	36	31	19	14
006	14	26	14	7	23	43	23	11	17	18	4	6	38	40	9	13
007	13	39	18	5	17	52	24	7	19	16	11	3	39	33	22	6
008	20	14	7	3	45	32	16	7	12	14	7	4	32	38	19	11
009	18	14	6	5	42	33	14	12	11	14	8	5	29	37	21	13
010	23	24	6	3	41	43	11	5	16	19	3	6	36	43	7	14
011	24	23	14	7	35	34	21	10	19	11	4	5	49	28	10	13
MEAN	18	22	10	5	35	39	17	9	15	14	6	4	38	35	16	10
SD	5	8	5	2	11	6	6	4	5	4	3	1	9	10	6	3

https://doi.org/10.3390/jox16030092

Table S5. Distribution of MP size classes (count and percentage) by the ten volunteers involved in the control group (Mean ± SD)

Healt Volunt	Phase 1								Phase 2							
	**N. MP size (μm)**				**% MP size (μm)**				**N. MP size (μm)**				**% MP size (μm)**			
	**0 - 10**	**11 - 30**	**31 - 50**	**51 - 80**	**0 - 10**	**11 - 30**	**31 - 50**	**51 - 80**	**0 - 10**	**11 - 30**	**31 - 50**	**51 - 80**	**0 - 10**	**11 - 30**	**31 - 50**	**51 - 80**
012	21	18	6	3	44	38	13	6	22	18	4	2	48	39	9	4
013	16	29	8	4	28	51	14	7	21	26	4	5	38	46	7	9
014	18	16	10	4	38	33	21	8	19	16	7	5	40	34	15	11
015	24	25	18	5	33	35	25	7	28	25	17	4	38	34	23	5
016	19	24	8	8	32	41	14	14	17	29	10	7	27	46	16	11
017	22	25	10	7	34	39	16	11	24	24	14	6	35	35	21	9
018	24	21	19	5	35	30	28	7	29	27	10	6	40	38	14	8
019	28	24	17	4	38	33	23	5	29	24	11	5	42	35	16	7
020	23	29	12	7	32	41	17	10	31	29	14	2	41	38	18	3
021	19	28	14	5	29	42	21	8	19	29	11	6	29	45	17	9
MEAN	21	24	12	5	34	38	19	8	24	25	10	5	38	39	16	8
SD	4	4	5	2	5	6	5	2	5	5	4	2	6	5	5	3

https://doi.org/10.3390/jox16030092

Table S6. Type and frequency of MPs in each volunteer involved in the cohort study

MP Type	Healthy Volunteer																						Frequency (N.)
	001		002		003		004		005		006		007		008		009		010		011		
	Phase 1	Phase 2	Phase 1	Phase 2	Phase 1	Phase 2	Phase 1	Phase 2	Phase 1	Phase 2	Phase 1	Phase 2	Phase 1	Phase 2	Phase 1	Phase 2	Phase 1	Phase 2	Phase 1	Phase 2	Phase 1	Phase 2	
ABS	6	4	-	-	4	2	-	-	4	1	-	-	5	2	-	-	-	-	4	3	-	-	5
BBP	-	-	-	-	-	-	-	-	5	4	4	4	8	6	-	-	3	3	4	4	5	3	6
CE	7	5	-	-	2	1	2	1	1	-	-	-	7	6	2	1	3	2	-	-	-	-	7
EPR	-	-	3	3	-	-	-	-	-	-	3	3	-	-	3	3	-	-	-	-	-	-	3
ER	5	4	-	-	-	-	-	-	-	-	-	-	-	-	-	-	-	-	3	1	4	3	3
PA	-	-	2	2	3	2	4	3	-	-	6	3	-	-	-	-	3	2	3	2	5	3	7
PAN	-	-	-	-	2	2	-	-	-	-	4	3	6	5	1	1	4	3	5	4	4	2	7
PBA	-	-	6	5	2	2	2	2	-	-	3	2	-	-	-	-	4	3	-	-	3	3	6
PC	-	-	7	6	3	2	1	1	4	2	6	5	3	3	2	2	2	-	-	-	4	2	9
PDMS	-	-	4	3	-	-	1	1	6	5	5	4	4	3	-	-	-	-	5	4	5	4	7
PE	15	7	8	5	3	2	6	4	11	7	12	8	10	5	8	6	7	3	11	8	13	7	11
PES	11	9	3	2	5	4	7	6	-	-	-	-	-	-	6	5	4	4	8	6	6	3	8
PET	7	6	4	3	5	3	6	5	5	4	-	-	8	5	5	4	4	4	5	3	6	4	10
PP	7	6	5	4	3	2	5	4	4	2	4	3	7	4	6	3	6	5	6	3	8	3	11
PS	-	-	6	4	2	2	3	3	4	3	7	5	4	3	-	-	-	-	-	-	-	-	6
PTFE	-	-	3	3	-	-	3	3	6	4	7	5	4	2	4	3	-	-	-	-	-	-	6
RA	11	8	-	-	2	2	-	-	7	4	-	-	9	4	7	6	3	3	2	-	5	2	8
TOTAL	69	49	51	40	36	26	40	33	57	36	61	45	75	48	44	34	43	32	56	38	68	39	-

Table S7. Type and frequency of MPs in each volunteer involved in the control group

MP Type	Healthy Volunteer 012		013		014		015		016		017		018		019		020		021		Frequency (N.)
	Phase 1	Phase 2	Phase 1	Phase 2	Phase 1	Phase 2	Phase 1	Phase 2	Phase 1	Phase 2	Phase 1	Phase 2	Phase 1	Phase 2	Phase 1	Phase 2	Phase 1	Phase 2	Phase 1	Phase 2	
ABS	4	4	-	-	-	-	5	4	4	4	-	-	4	6	5	4	-	-	4	4	6
BBP	-	-	5	6	-	-	-	-	4	5	3	1	4	4	3	2	-	-	-	-	5
CE	4	5	6	4	-	-	-	-	6	8	-	-	5	4	5	4	-	-	-	-	5
EPR	4	4	-	-	-	-	4	5	3	3	3	1	-	-	2	2	-	-	6	2	6
ER	3	3	-	-	-	-	-	-	-	-	-	-	-	-	-	-	-	-	-	-	1
PA	1	1	7	8	6	5	11	10	-	-	7	8	-	-	-	-	7	9	6	5	7
PAN	-	-	-	-	5	5	-	-	-	-	6	7	-	-	3	3	5	5	-	-	4
PBA	-	-	4	3	4	4	6	7	-	-	4	4	-	-	-	-	5	7	4	6	6
PC	-	-	4	3	3	3	3	3	-	-	4	4	7	6	6	5	5	7	5	2	8
PDMS	-	-	-	-	-	-	4	3	4	5	6	4	5	8	-	-	5	4	3	3	6
PE	8	7	11	12	9	11	14	14	13	14	13	16	14	18	3	3	15	16	17	19	10
PES	7	6	6	4	8	8	-	-	-	-	6	6	-	-	14	12	5	4	-	-	6
PET	8	8	7	8	7	7	10	12	12	8	-	-	9	7	11	15	7	4	9	11	9
PP	-	-	4	6	3	4	4	4	9	8	4	7	9	11	9	7	5	6	12	13	9
PS	-	-	3	3	3	-	5	5	-	-	5	6	5	4	-	-	6	8	-	-	6
PTFE	-	-	-	-	-	-	3	5	3	6	3	4	2	2	5	5	-	-	-	-	5
RA	9	8	-	-	-	-	3	2	1	2	-	-	5	2	7	7	6	6	-	-	6
TOTAL	48	46	57	57	48	47	72	74	59	63	64	68	69	72	73	69	71	76	66	65	-

Table S8. Studies on MPs in human blood in the literature

Location	N. volunteers	MP concentration	Type of MPs	Shape	Size (µm)	Analytical Technique	Reference
Pescara, Italy	11	1.40 - 2.23 µg/mL	ABS, BBP, CE, EPR, ER, PA, PAN, PBA, PC, PDMS, PE, PES, PET, PP, PS, PTFE, RA	Fragments	1 - 80	Stereomicroscope, SEM, µFTIR	Present study, 2026
Mohammed Seddik Ben Yahya Hospital, Jije, Algeria	38	-	PP, PET	Fragments, fibres, microbeads, films	50 - 300	Stereomicroscope, µFTIR	[61]
Guangdong, China	20	121 – 152 µg/mL	PA, PBAT, PC, PE, PET, PLA, PMMA, PP, PS, PVC	-	-	Py-GC-MS	[62]
Amsterdam, Netherlands	102	413 - 827 ng/mL	PE, PET, PMMA, PP, PS, PVC	Fragments	-	Py-GC-MS	[63]
Zhejiang Province, China	229	1 - 39 µg/mL	PA, PE, PET, PMMA, PP, PS, PVC	-	-	Py-GC-MS	[4]
First Affiliated Hospital of Guangzhou Medical University, China	33	1 - 50 µg/g	ACR, PA, PBAT, PC, PE, PET, PMMA, PP, PS, PTFE, PU, PVC	Fragments, fibres, granulars	-	SEM, LDIR, Py-GC-MS	[6]
Amsterdam, Netherlands	68	170 - 2,490 ng/mL	PA, PE, PET, PMMA, PP, PS, PVC	-	-	Py-GC-MS	[16]
Inha University Hospital, South Korea	36	4.2 MPs/mL	PA, PE, PET, PP, PS	Fragments, fibres	5 - 100	SEM, µFTIR, µRaman	[2]
Centre of Biomedicine, University of Hull, United Kingdom	20	1.84 – 4.65 µg/mL	AC, EAA, EBC, EPDM, EPM, EVA, EVA/EVOH, PA, PBI, PE, PEA, PET, PDMS, PHD, PO, POM, PP, PPA, PUR, PVC, Resin, VSC	Fragments, fibres	5 - 800	µFTIR	[11]
Shanghai Institute of Maternal-Fetal Medicine and Gynecologic Oncology, Shanghai, China	12	2 - 10 MPs/g	ACR, BR, EVA, FKM, MBS, PE, PET, PLA, PMMA, PP, PS, PU, PVC	-	20 - 100	LDIR	[31]
Nanjing Medical University, Nanjing, China	104	-	PA, PE, PEAA, PSAN, PVAL	Fragments, fibres	20 - 67	µRaman	[64]

Table S8. *Continued*

https://doi.org/10.3390/jox16030092

Barcelona, Spain	73	88–1,460 events/µL)	-	Fragments, fibres	≥ 2	Flow cytometry	[65]
Capital Medical University, Beijing, China	53	2 - 114 MPs/mL	PA, PC, PE, PET, PMMA, PP, PS, PU, PVC	-	20 - 184	LDIR	[66]
Amsterdam, Netherlands	22	1 - 13 µg/mL	PE, PET, PMMA, PS	Fragments	≥ 0.7	Py-GC-MS	[3]
Canakkale Onsekiz Mart University, Türkiye	3	25 - 500 µg/mL	PE	Microbeads	10 - 45	Cytokinesis-block micronucleus cytome (CBMN) assay	[67]

https://doi.org/10.3390/jox16030092

Written informed consent model

CONSENSO INFORMATO PER L'UTILIZZO DI CHITOSANO PER LA RIDUZIONE DELLE MICROPLASTICHE NEL SANGUE

Io sottoscritto/a _______________, nato/a il _______________, residente in _______________, dichiaro di aver ricevuto e compreso le informazioni relative all'utilizzo del prodotto a base di chitosano per la riduzione delle microplastiche nel sangue, per la futura pubblicazione dell'articolo "*Reduction of Circulating Microplastics in Humans Following Gastrointestinal Sequestration by Chitosan: A Pilot Controlled Study*"

Descrizione del prodotto

Il chitosano è una sostanza naturale derivata dalla chitina, che si può usare come Food Supplement, che ha dimostrato la capacità di legare le microplastiche presenti nell'intestino, facilitandone l'eliminazione attraverso le feci.

Benefici attesi

- Possibile riduzione della presenza di microplastiche nell'organismo
- Supporto alla salute intestinale
- Potenziale miglioramento del benessere generale

Possibili rischi e effetti collaterali

- Possibile insorgenza di disturbi digestivi (gonfiore, stipsi, diarrea)
- Reazioni allergiche in soggetti sensibili ai derivati della chitina
- Interferenze con l'assorbimento di alcuni nutrienti o farmaci

Condizioni d'uso

- Assunzione secondo le indicazioni del produttore o di un professionista sanitario
- Segnalazione immediata di eventuali effetti avversi
- Non adatto a soggetti allergici ai crostacei

Sono consapevole che io/il paziente non riceverò alcun beneficio economico o compenso dalla pubblicazione dell'articolo. L'uso/riutilizzo del mio materiale può includere (a titolo esemplificativo ma non esaustivo) la pubblicazione, la condivisione e l'adattamento del materiale in edizioni cartacee ed elettroniche delle riviste MDPI, su siti web, in edizioni concesse in sublicenza o ristampate (incluse le edizioni in lingua straniera) e in altre opere o prodotti, purché venga fornito il dovuto accreditamento/citazione della pubblicazione originale.

Privacy e trattamento dati

I dati raccolti in questo consenso saranno trattati nel rispetto delle normative vigenti sulla privacy (Regolamento UE 2016/679 - GDPR). La firma di questo modulo non pregiudica il mio diritto alla privacy, garantito dalla legge, né il diritto del paziente alla privacy. Sono consapevole di poter revocare il consenso in qualsiasi momento prima della pubblicazione, ma dopo la pubblicazione il mio consenso non potrà più essere revocato.

Dichiaro di aver ricevuto tutte le informazioni necessarie, di aver avuto l'opportunità di porre domande e di accettare consapevolmente l'uso del prodotto.

Firma: _______________ **Data:** _______________

https://doi.org/10.3390/jox16030092

Figure S4: Ethics Committee Approval

Ethics Committee Approval

DECISIONE COMITATO

In data 16 Settembre 2025 Il Comitato Etico Scientifico di JAPS

del Centro di Ricerca Clinica dell'Università Gabriele d'Annunzio di Pescara approva il protocollo numero MNP. A1

Il presidente della commissione

Prof Agostino Consoli

Translation

The trials have been approved by the International Agency for Pharma Standard Supplements (IAPS) in Pescara, Italy. The code of ethics of experiments that the scientific committee has listed on the approval date of 16 September 2024; is the following: Code MNP-A1.

https://doi.org/10.3390/jox16030092

Figure S5. Stereomicroscope images of MPs identified in this study: from (a-c) MPs in Phase 1 samples; from (d-f) MPs in Phase 2 samples (in cohort study and control group).

a)

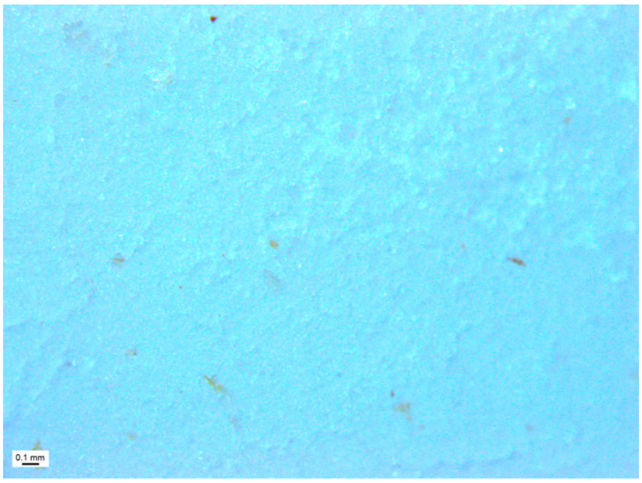

b)

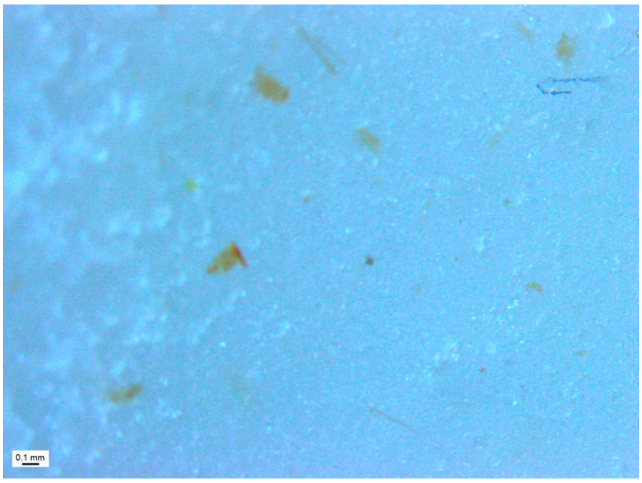

c)

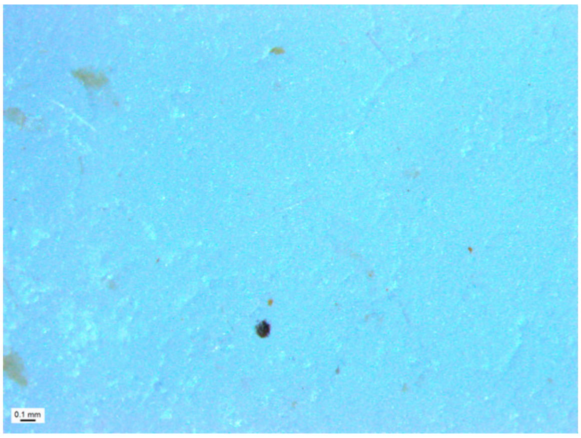

https://doi.org/10.3390/jox16030092

d)

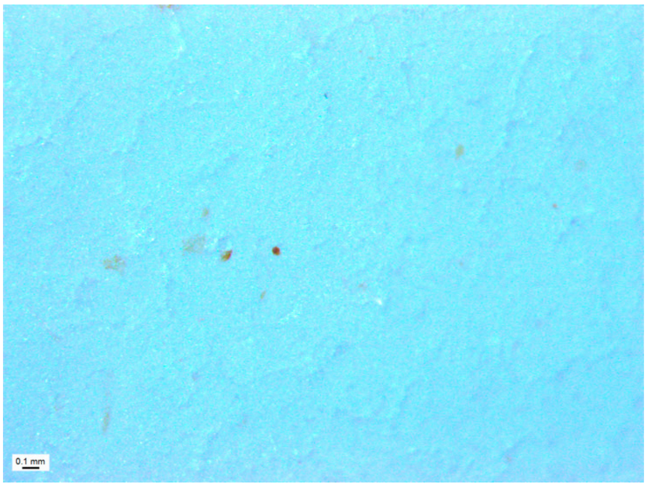

e)

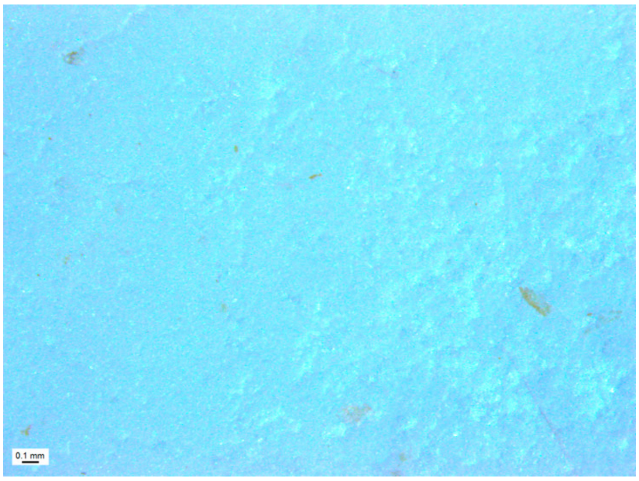

f)

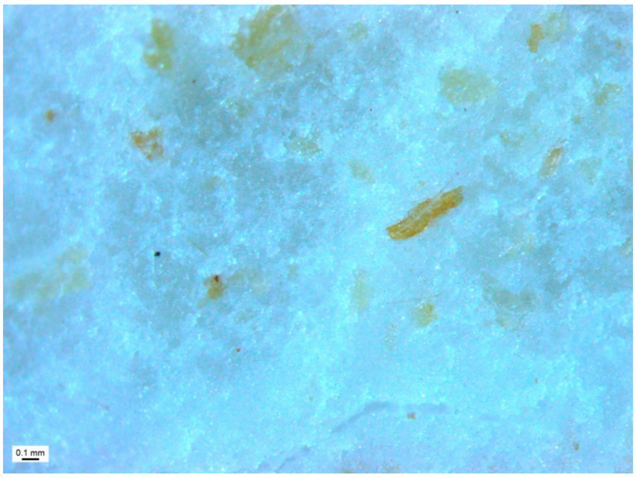

Figure S6. Illustrative SEM images of MPs identified in the blood samples, in the present study.

a)

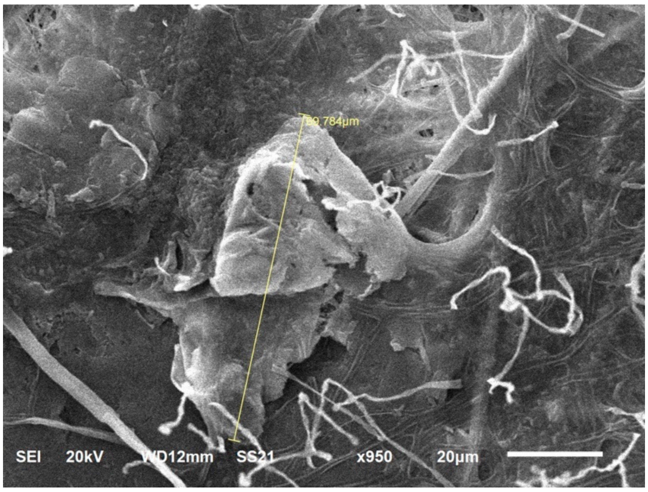

b)

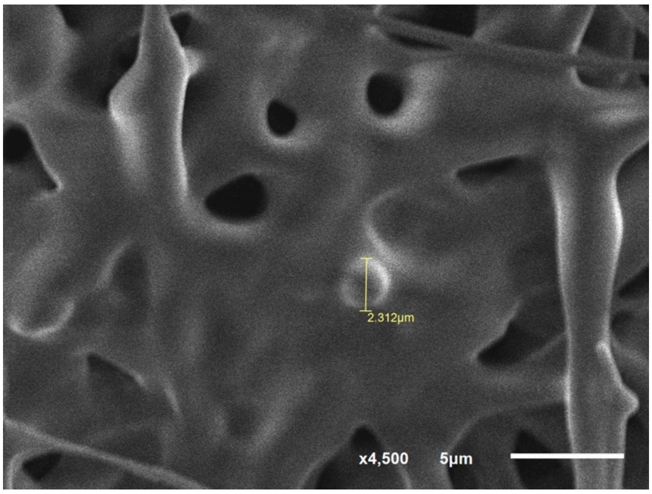

https://doi.org/10.3390/jox16030092

c)

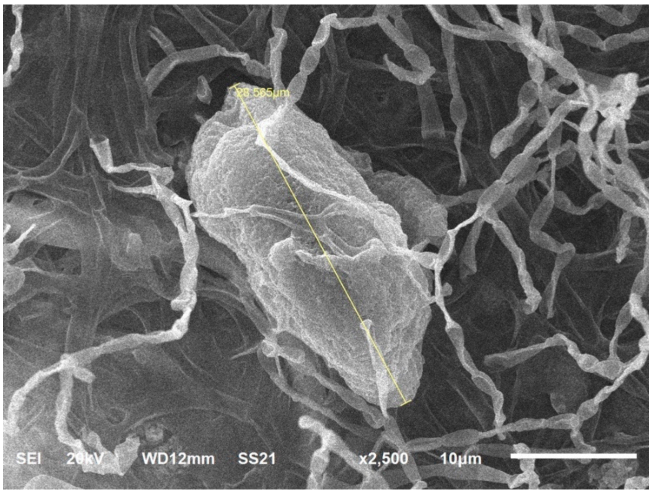
SEI 20kV WD12mm SS21 x2,500 10µm

d)

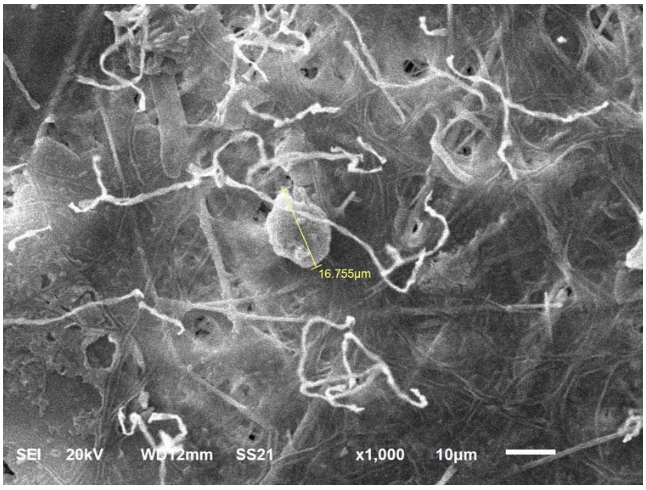
16.755µm
SEI 20kV WD12mm SS21 x1,000 10µm

e)

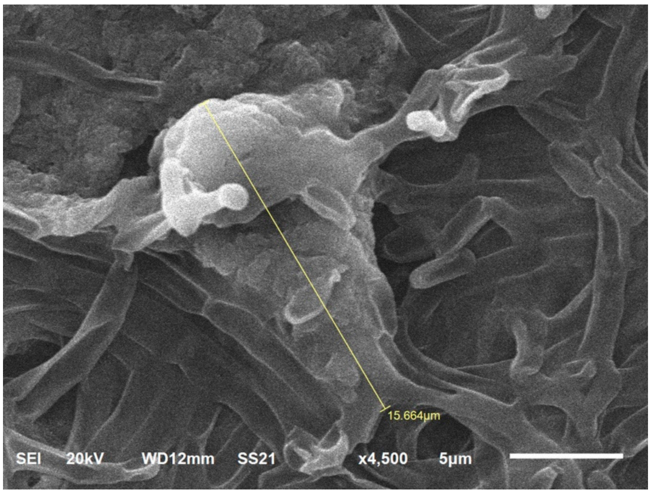
15.664µm
SEI 20kV WD12mm SS21 x4,500 5µm

f)

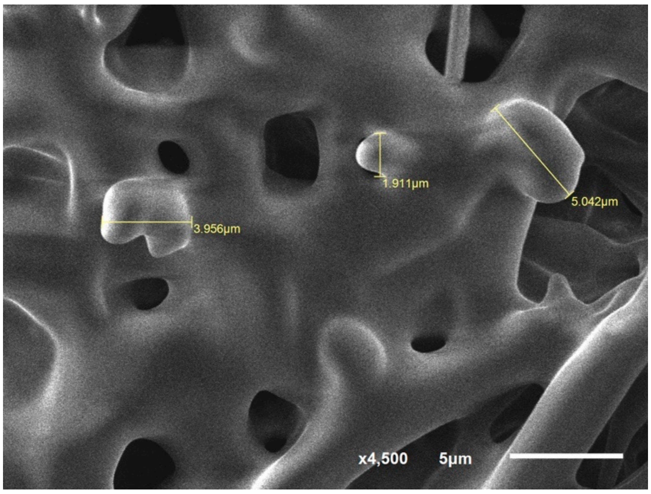

g)

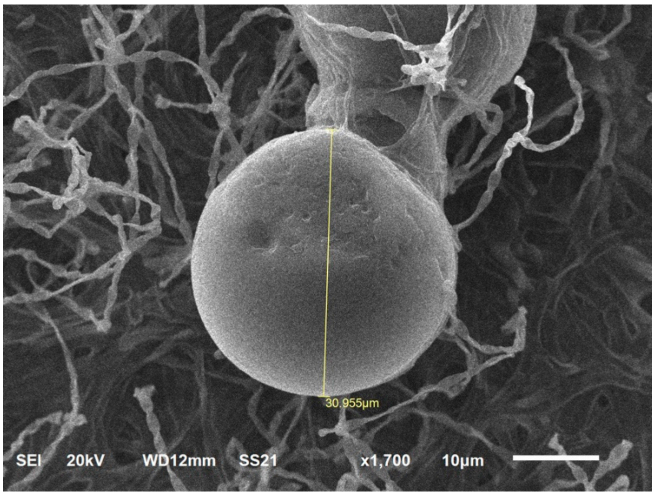

h)

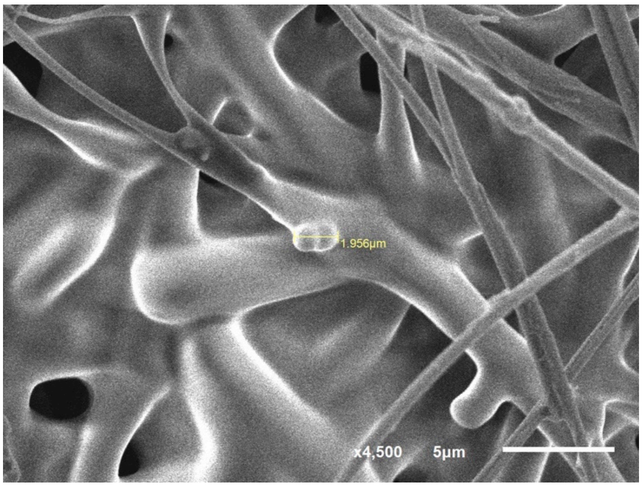

https://doi.org/10.3390/jox16030092

i).

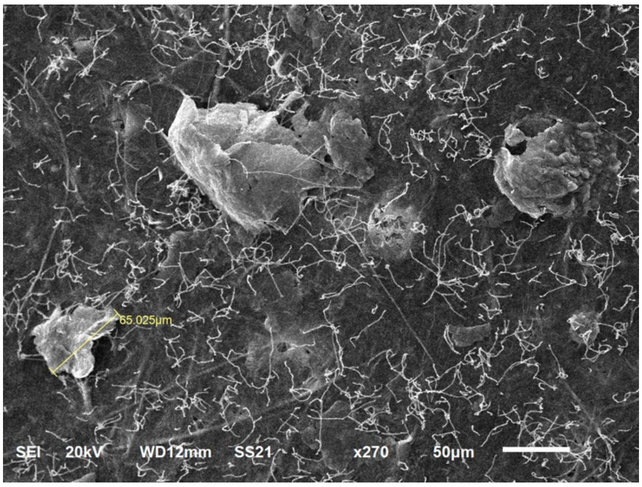

Supplement: Supplementary file 1 [file jox-16-00092-s001.zip › jox-4286784-supplementary.pdf]
